# A Porous Metal–Organic Framework Assembled by [Cu_30_] Nanocages: Serving as Recyclable Catalysts for CO_2_ Fixation with Aziridines

**DOI:** 10.1002/advs.201600048

**Published:** 2016-05-17

**Authors:** Hang Xu, Xiao‐Fang Liu, Chun‐Shuai Cao, Bin Zhao, Peng Cheng, Liang‐Nian He

**Affiliations:** ^1^ Department of ChemistryKey Laboratory of Advanced Energy Material ChemistryNankai UniversityTianjin300071China; ^2^ Collaborative Innovation Center of Chemical Science and Engineering (Tianjin)Tianjin300071China; ^3^ State Key Laboratory and Institute of Elemento‐Organic ChemistryNankai UniversityTianjin300071China

**Keywords:** aziridines, carbon dioxide fixation, metal–organic frameworks, nano‐sized cages, regenerable heterogeneous catalysis

## Abstract

Based on a novel ligand 5‐(2,6‐bis(4‐carboxyphenyl)pyridin‐4‐yl)isophthalic acid (H_4_BCP) with large skeletons, a unique porous framework {[Cu_2_(BCP)(H_2_O)_2_]·3DMF}*_n_* (**1**) assembled by nano‐sized and censer‐like [Cu_30_] cages is successfully obtained and structurally characterized. In **1**, the large 1D channel in frameworks and window size in the nanocages can enrich methylene blue and capture CO_2_, exhibiting the promising applications in environmental protection. More importantly, the explorations on the cycloaddition reaction of CO_2_ and aziridines with various substituents suggest that **1** can serve as an efficient heterogeneous catalyst for CO_2_ conversion with aziridines in a solvent‐free system, which can be reused at least ten times without any obvious loss in catalytic activity. This is the first example of metal–organic framework (MOF)‐based catalysts in converting CO_2_ into high‐value oxazolidinones through activating aziridines and CO_2_, further extending the applications of MOFs materials in catalysis.

## 1 Introduction

Environment and energy are crucial issues in our industrialized age, and excessive depletion of fossil energy would generate a large number of pollutants, resulting in a series of social and environmental problems. Carbon dioxide (CO_2_), as the major waste gas from industrial and human activities, can be considered as one of the main greenhouse gases responsible for global warming.^1^ Hence, the effective utilization associated with reduction of carbon emission has become an urgent and challenging research topic,^2^ and many efforts have been directed toward converting CO_2_ into various valuable chemicals,^3^ which are currently obtained from fossil fuel resources. One of the effective strategies for CO_2_ conversion is the coupling reaction of CO_2_ with aziridines to afford oxazolidinones due to not only high reactivity of aziridines toward CO_2_ and ideal atom efficiency, but also the important applications of oxazolidinones as chiral auxiliaries, intermediates in organic synthesis, and medical chemistry.^4^ In such type of reactions, many homogeneous catalysts, such as α‐amino acids, alkali metal halide, and tetraalkylammonium salt, have been investigated and exhibit good activity,^5^ but comparably, only a few heterogeneous catalysts were employed in this reaction.^6^ Therefore, to approach practical applications, it is of key importance to design and synthesize efficient and heterogeneous catalysts for CO_2_ conversion.

On the other hand, porous metal–organic frameworks (MOFs) can be considered as a promising candidate for the catalytic reaction as they have designable structures and modifiable functional groups, well‐defined pores and high surface area, significant capability for CO_2_ adsorption and excellent recyclable catalytic performance.^7^, ^8^, ^9^, ^10^ The utilization of MOFs as catalysts for CO_2_ conversion therefore has received tremendous attention, and several MOFs have already shown high catalytic activity in CO_2_ conversion with the terminal alkyne activation, the hydroboration and cycloaddition of epoxides.^11^ However, to the best of our knowledge, MOFs‐based catalysts for CO_2_ conversion with aziridines still have not been reported so far.

In this contribution, we designed and synthesized a novel low symmetry organic ligand 5‐(2,6‐bis(4‐carboxyphenyl)pyridin‐4‐yl)isophthalic acid (H_4_BCP, Scheme S1, Supporting Information). Then, a unique 3D framework {[Cu_2_(BCP)(H_2_O)_2_]**·**3DMF}*_n_* (**1**) assembled by [Cu_30_] nanocages was obtained and structurally characterized. Importantly, in the cycloaddition reaction of CO_2_ and aziridines, **1** can act as a highly effective and recoverable catalyst, which can be reused at least ten times without any obvious loss. To our knowledge, it represents the first example of MOFs‐based catalyst for CO_2_ conversion with aziridines.

## 2 Results and Discussion

**Figure 1:**
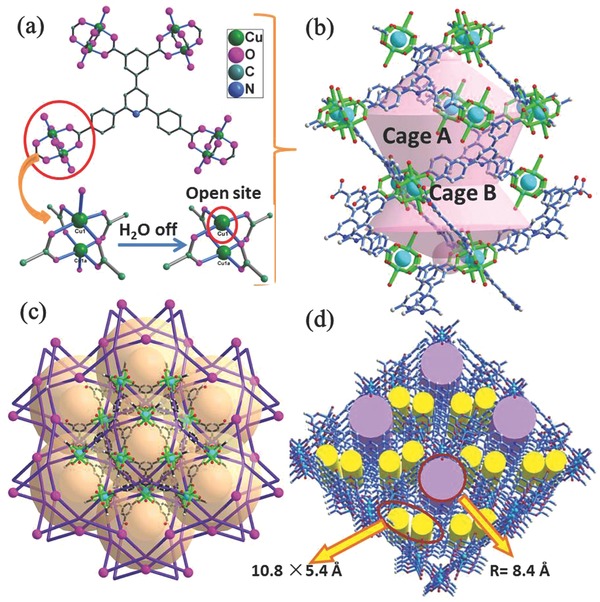
a) The coordination environments of Cu^2+^ and BCP^4−^ ligand. b) Nano‐sized [Cu_30_] cage in 1. c) Perspective view of the framework of 1 along the *c*‐direction. d) 3D framework and 1D channels along the *b*‐direction.

Large organic ligand H_4_BCP with *C*_2_ symmetry was first synthesized, and the corresponding molecule structure was characterized by single crystal X‐ray diffractions (Table S1, Supporting Information). Based on H_4_BCP ligand, the green crystals of **1** were obtained by solvothermal methods, and the structural analyses revealed that compound **1** belongs to the trigonal system with space group *R*3m (Table S1, Supporting Information). The 3D framework of **1** is assembled by the unique nano‐sized [Cu_30_] cages (**Figure** **1**b), which are constructed from 15 Cu_2_(O_2_CR)_4_ paddlewheel units and BCP linkers. The shape of [Cu_30_] cage looks like a distorted censer (Figure S1, Supporting Information), which is distinctive to the reported polyhedral/spherical cages in classic MOFs, for instance, eight [Zn_4_O] units as vertexes construct a cubic cage in MOF‐5, 24 Zn atoms and 48 methylimidazole linkers form a spherical cage in ZIF‐8, etc.^12^, ^13^, ^14^, ^15^, ^16^, ^17^, ^18^, ^19^, ^20^ Each [Cu_30_] cage can be divided into two subcages, big cage A and small cage B (Figure 1b) to describe in details. The cage A is established by 12 Cu_2_(O_2_CR)_4_ paddlewheel units and six BCP^4−^ anions, displaying a distorted hexagonal bipyramid. Both top and bottom in cage A are occupied by three Cu_2_(O_2_CR)_4_ paddlewheel units with the window of 8.1 and 16 Å (Figure S1, Supporting Information), respectively. The middle part of cage A contains six Cu_2_(O_2_CR)_4_ paddlewheel units with apertures of ≈21 Å (Figure S1, Supporting Information). The cage B shares with three Cu_2_(O_2_CR)_4_ paddlewheel units from the bottom of cage A, and six BCP linkers coordinate to another three Cu_2_(O_2_CR)_4_ units into a hexagonal bipyramid with a pore size of 21 Å (Figure S1, Supporting Information). Along the *b*‐axis and *c*‐axis directions, compound **1** exhibits irregular 1D channels, and the total potential pore volume is as high as 72.1% calculated by the PLATON program.

From the topological point of view, each paddlewheel unit of Cu_2_(O_2_CR)_4_, linking with four BCP^4−^, can be considered as a 4‐connected node; and each BCP^4−^ anion bridges four Cu_2_(O_2_CR)_4_ unit, thus it also can be regarded as a 4‐connected node. On the basis of this simplification, the resulting network of compound **1** can be described as a (4, 4)‐connected framework with the *nbo*‐type topology.

**Figure 2:**
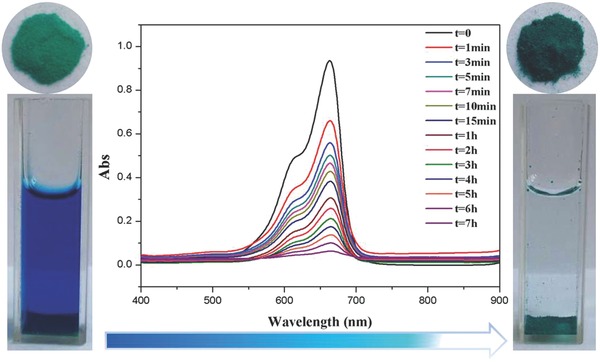
The UV spectra and color change after absorbing MB.

Considering the large window size and cavity in [Cu_30_] cage, the exploration on the sorption of methylene blue (MB) was carried out. First, 20 mg compound **1** was immersed into the MB solution, and the concentration of MB was monitored by UV–vis spectrophotometer. The results revealed that the concentration of MB gradually decreased as the increased time, and MB in the solution was almost completely absorbed after 7 h with the color change from dark blue to colorless (**Figure** **2**). The total adsorption of MB can reach up to 205 mg g^−1^, the high adsorption capacity of MB clearly confirmed the large cages and windows in **1**. The results implied that large substrate molecules can enter into the cage, and **1** can provide enough space to conduct the cycloaddition reaction of CO_2_. Additionally, TGA and CO_2_ sorption of compound **1** were also investigated (Figures S4 and S6, Supporting Information), being indicative of that porous framework keeps intact until about 270 °C and possesses the capacity of capturing CO_2_. Therefore, porous material **1** with open Cu^2+^ as Lewis acidic sites displays the promising application for the coupling reaction of CO_2_ with aziridines.

In order to explore the optimized reaction condition, the reactions of CO_2_ with aziridines under various conditions were investigated (**Table** **1**). In the preliminary study, 1‐ethyl‐2‐phenylaziridine (**1a**) was selected as a model substrate to investigate the influence of temperature on the reaction (Table 1, entries 1–4). At room temperature, the corresponding oxazolidinones of 3‐ethyl‐5‐phenyloxazolidin‐2‐one (**2a**) and 3‐ethyl‐4‐phenyloxazolidin‐2‐one (**3a**) were obtained with only 30% yield, and then a significant increase in the yield was observed as temperature increased to 80 °C, indicating that higher temperature can facilitate the cycloaddition reaction. Quantitative yields together with excellent selectivity toward **2a** were achieved at 100 °C. However, the yield at 120 °C decreased to 85%, which might be ascribed to the partial decomposition of co‐catalyst tetrabutylammonium bromide (TBAB) in high temperature. As a result, 100 °C is optimal temperature for this reaction, giving the best results. Then the influence of CO_2_ pressure and the amount of cat. **1** on the reaction were investigated at 100 °C. Under 1 MPa CO_2_, the yield decreased to 92% (entry 5), which was clearly inferior to the yield with 2 MPa CO_2_ (entry 3), indicative of that 2 MPa CO_2_ is a suitable pressure. In terms of catalyst amount, the yield decreased to 89% when the amount of **1** was decreased to 40 mg (entry 6). As a result, the optimized reaction conditions should be 100 °C under 2 MPa CO_2_ with 80 mg catalyst **1**. Then, to explore the catalytic performance of other porous MOFs with paddlewheel Cu_2_(O_2_CR)_4_ units, well‐known HKUST‐1 was selected as catalyst for this reaction. The corresponding yield of 3‐ethyl‐5‐phenyloxazolidin‐2‐one is 95% with good selectivity (Table 1, entry 11), indicating that both of two porous MOFs with paddlewheel Cu_2_(O_2_CR)_4_ units can act as good catalysts for the cycloaddition reaction of CO_2_ and aziridines. Comparing to HKUST‐1, compound **1** shows a little higher activity, possibly being attributed to the different cage‐like structure and larger porosity in compound **1**.

**Table 1:** Cycloaddition reaction of CO_2_ with 1‐ethyl‐2‐phenylaziridine under various conditionsa)

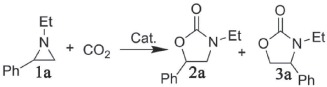				
Entry	Catalyst **1** [mg]	*T* [°C]	Yieldb) [%]	Regio‐selc)
1	80	25	30	97:3
2	80	80	91	97:3
3	80	100	>99	98:2
4	80	120	85	97:3
5d)	80	100	92	98:2
6	40	100	89	98:2
7e)	0	100	45	92:8
8f)	0	100	43	92:8
9g)	0	100	47	>99
10	0	100	79	98:2
11h)	40	100	95	96:4

^a)^Reaction conditions: 1‐ethyl‐2‐phenylaziridine (294.4 mg, 2.0 mmol), solvent‐free, catalyst **1**, TBAB (32.4 mg, 0.1 mmol), CO_2_ (2.0 MPa), 12 h, 80 mg catalyst **1** loading (based on metal center, about 10 mol%);

^b)^Total yield of **2a** and **3a** determined by ^1^H NMR using 1,3,5‐trimethoxybenzene as an internal standard;

^c)^Molar ratio of **2a** to **3a**;

^d)^CO_2_ (1.0 MPa);

^e)^TBAB (32.4 mg, 0.1 mmol) and Cu(OAc)_2_ (39.9 mg, 0.2 mmol);

^f)^TBAB (32.4 mg, 0.1 mmol) and Cu(NO_3_)_2_ (37.4 mg, 0.2 mmol);

^g)^TBAB (32.4 mg, 0.1 mmol) and CuSO_4_ (31.9 mg, 0.2 mmol);

^h)^HKUST replaces compound **1** to conduct this reaction, catalyst loading (based on metal center, about 10 mol%).

To further explore and exemplify the catalytic potential of compound **1**, several typical aziridines were prepared and subjected to the cycloaddition reaction (detailed characterization data of aziridines in the Supporting Information). Under the optimized reaction conditions, all of the corresponding oxazolidinones were obtained and summarized in **Table** **2** (entries 1–5). For entries 1–3, aziridines bearing three different groups at the nitrogen atom were tested in cycloaddition reaction. Interestingly, the aziridine with ethyl group on the nitrogen atom (entry 1) displayed the best reactive activity with CO_2_, possibly because of the relatively low steric hindrance, while other aziridines with larger alkyl groups (entries 2 and 3) were found to have lower catalytic activity. Additionally, keeping R^1^ as ethyl group, the different substituent groups on phenyl ring were also investigated on the reaction. The corresponding oxazolidinones from 1‐ethyl‐2‐(4‐chlorophenyl)‐aziridine and 1‐ethyl‐2‐(4‐methyl)‐aziridine were obtained with high yields (entries 4 and 5), but 1‐ethyl‐2‐(4‐chlorophenyl)‐aziridine exhibited relatively low activity among the aziridines tested, which might be attributed to the electron‐withdrawing effect of chloride group on the phenyl ring. The results clearly show that compound **1** possesses the capacity of catalyzing cycloaddition reaction with extensive aziridines.

**Table 2:** Synthesis of various oxazolidinones from CO_2_ and aziridines with catalyst 1a)

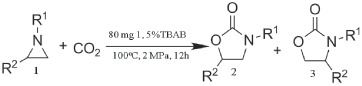				
Entry	Substrate	Conv.b) [%]	Yieldb) [%]	Regio‐selc)
1	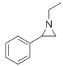	>99	>99	98:2
2	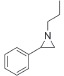	95	93	97:3
3	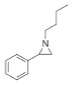	93	92	98:2
4	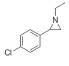	>99	88	93:7
5	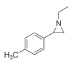	>99	92	98:2

^a)^Reaction conditions: aziridine (2.0 mmol), solvent‐free, 80 mg catalyst **1** loading (based on metal center, about 10 mol%), TBAB (32.4 mg, 0.1 mmol), CO_2_ (2.0 MPa), 12h;

^b)^Determined by ^1^H NMR using 1,3,5‐trimethoxybenzene as an internal standard;

^c)^Molar ratio of **2** to **3**.

**Figure 3:**
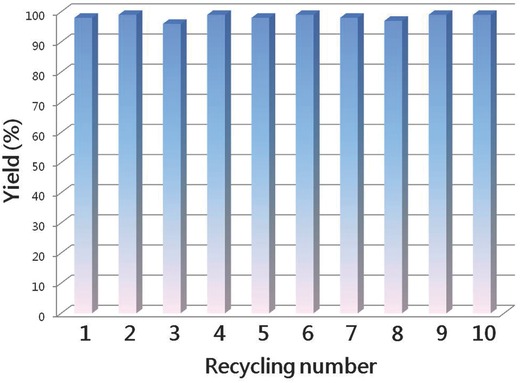
Recycle tests with 1 for the cycloaddition reaction of CO_2_ with 1‐ethyl‐2‐phenylaziridine.

Actually, the recyclable performance is an important parameter for heterogeneous catalyst, and compound **1** could be easily separated from the reaction mixture by centrifugation and filtration. Hence, the investigations on the recycle performance of **1** as catalysts were carried out. As shown in **Figure** **3**, the catalyst can be reused at least ten times without any obvious loss in catalytic activity, indicative of that **1** is more close to practical applications. The powder X‐ray diffraction (PXRD) patterns of used compound **1** revealed that **1** still keeps crystalline state after five cycles and has been transformed into amorphous state after the sixth run (Figure S7, Supporting Information) as following reasons: (a) Generally, the long‐range ordering crystalline state of crystals would lose after drastically stirring, and some peaks in PXRD would gradually broaden and/or weaken. In terms of compound **1**, the peaks in PXRD do not disappear after one time repeated reaction, and the intensity of peaks in PXRD gradually decreased after the recycle reaction (Figure S7, Supporting Information), suggesting that the crystalline state of **1** gradually becomes poor and results in amorphous state. (b) The ICP analyses of reaction filtrate revealed that only the trace amount leakage of Cu^2+^ was observed (Table S2, Supporting Information), indicating that the framework did not decompose after the repeated reaction. (c) X‐ray photoelectron spectroscopy (XPS) spectrum of the samples after ten times repeated reaction showed that oxidation state of Cu(II) keeps stable in framework **1** (Figure S8, Supporting Information). Therefore, we considered that the framework of **1** gradually transfers from crystalline to amorphous state, and framework is still stable. Thus, the stable framework in amorphous state still shows the well catalytic performance.

Next step, to investigate the reaction mechanism, the different copper sources were employed in the CO_2_ fixation reaction as control experiments. First, Cu(OAc)_2_, Cu(NO_3_)_2_, and CuSO_4_ as catalysts were chosen to replace compound **1** to conduct the reaction, and the yield was just 45%, 43%, and 47%, respectively (Table 1, entries 7–9). The results indicate that free Cu^2+^ ions did not effectively catalyze the cycloaddition reaction with 1‐ethyl‐2‐phenylaziridine, and the anions in salts also do not have obvious effect on the reaction. To our surprise, the yield of the reaction with TBAB (Table 1, entry 10) is higher than that under Cu‐salts and TBAB, which mainly originates from the binding effect between free Cu^2+^ and Br^−^ to hinder the nucleophilicity of Br^−^ ions.^21^ Comparably, the yield is over 99% when **1** serves as catalysts. The result clearly shows that Cu^2+^ in the framework of **1** can enhance over 50% catalytic capacity than isolated Cu^2+^ ions in this reaction. The significant enhancement of catalytic performance maybe benefit from the following factors: (a) 1D channels and nano‐sized [Cu_30_] cages in **1** can enrich or capture CO_2_ and substrate molecules, i.e., aziridines, and the increased concentration of reactants can accelerate the reaction; (b) the confinement effect of nano‐sized [Cu_30_] cages can enhance reactivity with aziridines and CO_2_; (c) in the stable Cu_2_(O_2_CR)_4_ paddlewheel unit, the steric hindrance around Cu^2+^ and electric neutrality of the unit effectively impede the coordination between Cu^2+^ and Br^−^, resulting in more nucleophilic attack of Br^−^ on aziridines. Namely, in effectively catalyzing the cycloaddition reaction with aziridines and CO_2_, both the unique coordination environments of Cu^2+^ and cage‐like motif in [Cu_30_] nanocages play a vital role.

Therefore, a possible mechanism for the 1/TBAB‐catalyzed cycloaddition of CO_2_ with aziridines is proposed. First, the substrate and CO_2_ are enriched in the nano‐sized [Cu_30_] cages. Then, the coupling reaction is initiated by coordination of copper site in compound 1 with the nitrogen atom of the aziridine. Second, the nucleophilic attack of Br^−^ can lead to the ring opening of the aziridine through two different pathways as represented by path (a) or (b) (Scheme S2, Supporting Information). The Cu_2_(O_2_CR)_4_ paddlewheel units in 1 can stabilize the obtained carbamate salt, which is converted into the corresponding oxazolidinone through intramolecular ring‐closure. In terms of selectivity, the main product 2a originates from ring‐opening of the aziridine at the most substituted carbon, which produced the more stable carbamate salt intermediate. So in most cases, the regioselectivity (molar ratio of carbamate salt) exceeds 90:10. Thus, the corresponding oxazolidinone is obtained with high yield and selectivity under 1 as the catalyst.

## 3 Conclusion

In conclusion, a unique porous framework **1** with 30‐nuclear copper nanocages was synthesized and structurally characterized, possessing the capacity of capturing MB and CO_2_, which shows the promising applications in environmental protection field. Importantly, compound **1** was demonstrated as a new type of heterogeneous catalyst for CO_2_ fixation, acting as an efficient, recyclable, and environmentally friendly material to catalyze the cyclization reaction with aziridines and CO_2_ in a solvent‐free system. It should be noticed that robust catalyst **1** can be reused at least ten times without any obvious loss in catalytic activity. To our knowledge, it is the first example of MOF‐based catalysts for converting CO_2_ into oxazolidinones through activating aziridines, which expands the potential applications of MOFs in areas of catalysis and the reduction of CO_2_.

## 4 Experimental Section

*Synthesis of {[Cu_2_(BCP)(H_2_O)_2_]·3DMF}_n_ (* ***1*** *)*: A mixture of Cu(NO_3_)_2_ **·**3H_2_O (0.1 mmol, 24.1 mg), H_4_BCP (0.05 mmol, 24.2 mg), and HNO_3_ (100 μL) in DMF (5 mL) were sealed in a 10 mL Teflon‐lined stainless steel autoclave. The autoclave was heated at 100 °C for 3 d under autogenous pressure and then cooled to room temperature in 72 h. The green crystals were obtained and washed with DMF. Yield: 82%, based on H_4_BCP. Anal. calcd for compound **1**: C 50.13, H 4.41, N 6.50; Found: C 49.76, H 4.58, N 6.71.

*General Procedure for Cycloaddition of Aziridine and CO_2_*: In a 25 mL autoclave reactor equipped with a magnetic stirrer, aziridine (2 mmol), TBAB, and the MOF catalyst were first charged into the reactor. Then CO_2_ was introduced into the autoclave, followed by stirring at a predetermined temperature for 5 min to reach the equilibrium. The pressure was adjusted to the desired pressure and the mixture was stirred continuously for certain time. After the reaction was complete, the reactor was cooled in ice water and CO_2_ was ejected slowly. An aliquot of the sample was taken from the resulting mixture and dissolved in dry CH_2_Cl_2_ by adding 1,3,5‐trimethoxybenzene as an internal standard for ^1^H NMR analysis. The residue was purified by column chromatography on silica gel (200–300 mesh, eluting with 8:1 to 1:1 petroleum ether–ethyl acetate) to provide the desired products. The products were further identified by ^1^H NMR, ^13^C NMR, and MS, showing good agreement with the assigned structures and were consistent with those reported in the literature^22^ for the known compounds.

[CCDC 1442640 (**1**) and 1442639 (**H_4_BCP**) contain the supporting crystallographic data for this paper. These data can be obtained free of charge from The Cambridge Crystallographic Data Centre via www.ccdc.cam.ac.uk/data_request/cif.]

## Supporting information

As a service to our authors and readers, this journal provides supporting information supplied by the authors. Such materials are peer reviewed and may be re‐organized for online delivery, but are not copy‐edited or typeset. Technical support issues arising from supporting information (other than missing files) should be addressed to the authors.

Supplementary
