# Supplementary material for: A Porous Metal–Organic Framework Assembled by [Cu30] Nanocages: Serving as Recyclable Catalysts for CO2 Fixation with Aziridines
Source: Adv Sci (Weinh). 2016 May 17;3(11):1600048. doi: 10.1002/advs.201600048 (PMC5111788; doi:10.1002/advs.201600048)
Supplement: Supplementary file 1 — Supplementary [file ADVS-3-0g-s001.pdf]

Copyright WILEY-VCH Verlag GmbH & Co. KGaA, 69469 Weinheim, Germany, 2016.

ADVANCED SCIENCE
Open Access

Supporting Information

for *Adv. Sci.,* DOI: 10.1002/advs.201600048

A Porous Metal–Organic Framework Assembled by [Cu_{30}] Nanocages: Serving as Recyclable Catalysts for CO_2 Fixation with Aziridines

Hang Xu, Xiao-Fang Liu, Chun-Shuai Cao, Bin Zhao, Peng Cheng, and Liang-Nian He**

Copyright WILEY-VCH Verlag GmbH & Co. KGaA, 69469 Weinheim, Germany, 2013.

Supporting Information

A Porous Metal-Organic Framework Assembled by [Cu_{30}] Nano-Cages: Serving as Recyclable Catalysts for CO_2 Fixation with Aziridines

Hang Xu,[†] Xiao-Fang Liu,[†] Chun-Shuai Cao, Bin Zhao,* Peng Cheng *and* Liang-Nian He*

Experimental Section

Materials and methods

Novel organic ligand 5-(2,6-bis(4-carboxyphenyl)pyridin-4-yl)isophthalic acid (**H_4BCP**) with large skeleton was designed and successfully prepared by ourselves, and the corresponding details would be given as below. Various aziridines with different substituents were synthesized according to the reported methods,[1] and the corresponding process would be given in Scheme S3.

Other reagents and reactants were purchased commercially and were used directly without further purification. The C, H and N microanalyses were obtained at the Institute of Elemental Organic Chemistry, Nankai University. Powder X-ray diffraction and thermodiffractogram measurements were carried out on an Ultima Ⅳ X-ray diffractometer using Cu-Kα radiation. Thermogravimetric analyses (TGA) were recorded with a Netzsch TG 209 TG-DTA analyzer under a nitrogen atmosphere. Ultraviolet-visible (UV-vis) absorption spectra were recorded on a SHIMADZU UV-3600 UV-Vis-NIR spectrophotometer Isotherm of carbon dioxide was measured by a Quantachrome Autosorb-1 volumetric adsorption analyser. ^{1}H NMR spectra were recorded on a Bruker 300 or 400 spectrometer in $CDCl_3$ and $CDCl_3$ (7.26 ppm) was used as internal reference, ^{13}C NMR was recorded at 75 or 100.6 MHz in $CDCl_3$ and $CDCl_3$ (77.0 ppm) was used as internal reference. X-ray photoelectron spectroscopy (XPS) measurements were performed with a Kratos Axis Ultra DLD (delay-line detector) spectrometer equipped with a monochromatic Al-Kα X-ray source (1468.6 eV).

Preparation of 5-(2,6-bis(4-carboxyphenyl)pyridin-4-yl)isophthalic acid (H_4BCP).

Firstly, 3, 5-Dimethyl benzaldehyde (1.34 g, 10 mmol), 4-methylaceto phenone (3.35 g, 25 mmol) and power NaOH (1.2 g, 30 mmol) were crashed together with a pestle and mortar for 2 h, and orange powder was obtained. The orange powder was added to a stirred solution of ammonium acetate (8 g, excess) in ethanol (500 mL). The reaction mixture was heated at reflux for 10 h. After cooling to room temperature, a white precipitate was filtered, washed with water for three times and dried to afford the product. It was purified by recrystallization, and needle crystal (**DDTP**) was obtained. Yield (based on 3, 5-Dimethyl benzaldehyde): 2.97g, 85%.

Next step, **DDTP** (0.175g, 0.5 mmol), 2 mL HNO_3 and 8 mL H_2O were added to a 25 mL telfon-lined stainless steel container and heated to 180 °C for 12 h under autogenous pressure, then cooled to room temperature in 12 h. Block crystals were obtained and the yields are *ca.* 100%. ^{1}H NMR (400 MHz, d_6-DMSO) δ 13.37 (br, 4 H), 8.66 (s, 2 H), 8.56 (s, 1 H), 8.46 (d, ^3J = 8.464 H), 8.36 (s, 2 H), 8.09 (d, ^3J = 8.09, 4 H). ^{13}C NMR (100.6 MHz, d_6-DMSO) δ 167.58, 166.86, 156.17, 149.00, 142.71, 139.09, 132.73, 131.79, 130.97, 130.18, 127.65, 118.81.

Scheme S1

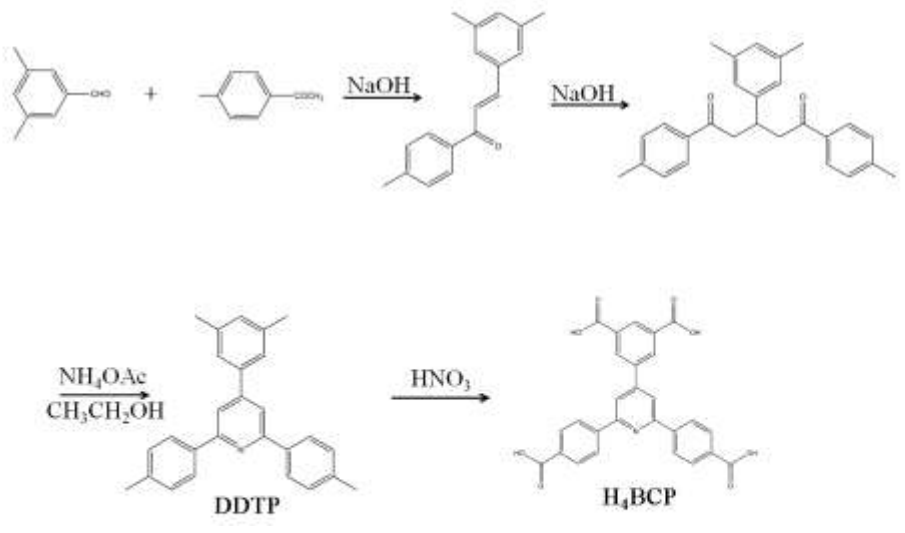

Scheme S2

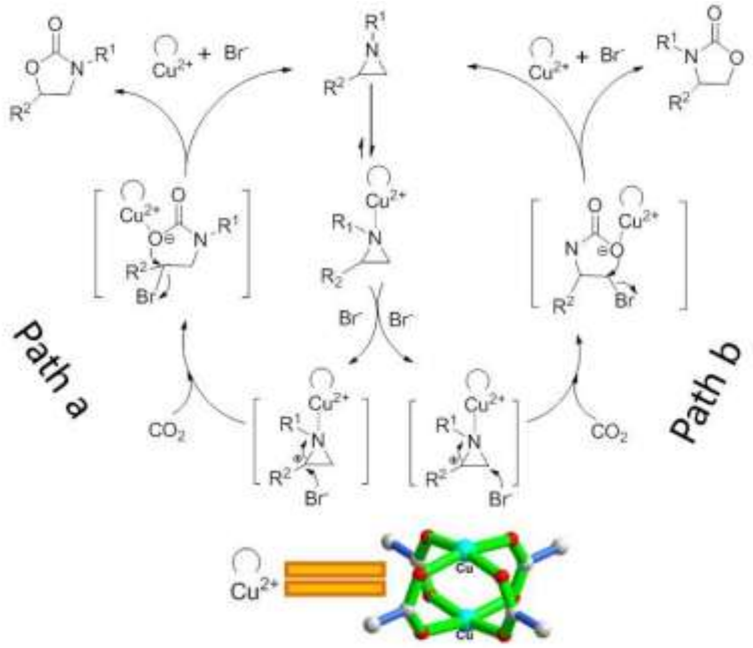

The possible mechanism for the MOF/TBAB-catalyzed cycloaddition of CO_2 with aziridine.

X-Ray crystallography

Suitable crystals **1** and H_4BCP were paced in a cooled N_2 gas stream at ~130 K for crystallographic data collection on a SuperNova Single Crystal Diffractometer equipped with graphite-monochromatic Mo-Kα radiation (λ = 0.71073 Å). Data reduction included absorption was performed by using the SAINT program.[2] The structures were solved by direct methods and refined by full-matrix least squares on F^2 with SHELXS-97 and SHELXL-97 programs.[3] All the hydrogen atoms were placed geometrically and refined using a riding model.

In terms of crystal **1**, we used PLATON/SQUEEZE[4] to remove the diffraction from the solvent region. The number of isolated DMF molecules was determined by TG analyses and elemental microanalyses. Detailed crystal data and structure refinement for **1** and **H_4BCP** are shown in Table S1.

Table S1 Crystals data and structure refinement for **1** and **H_4BCP**

Identification code	**1**	**H_4BCP**
CCDC Number	1442640	1442639
Empirical formula	$C_{36}H_{38}Cu_2N_4O_{13}$	$C_{27}H_{17}NO_8$
Formula weight	861.80	483.42
Temperature/K	130.15	121.4
Crystal system	trigonal	monoclinic
Space group	*R3m*	$P2_1/c$
a/Å	26.2928(18)	22.4340(7)
b/Å	26.2928(18)	7.0439(2)
c/Å	26.036(2)	13.5709(5)
α/°	90.00	90.00
β/°	90.00	103.787(3)
γ/°	120.00	90.00
Volume/Å^3	15588(2)	2082.74(12)
Z	9	4
F(000)	3996.0	1000.0
Goodness-of-fit on F^2	0.983	1.069
Final R indexes [I>=2σ (I)]	R_1 = 0.0612, wR_2 = 0.1509	R_1 = 0.0483, wR_2 = 0.1315
Final R indexes [all data]	R_1 = 0.0755, wR_2 = 0.1596	R_1 = 0.0601, wR_2 = 0.1408

Table S2 The ICP results of reaction filtrate.

Recycle Time	Cu^{2+} (ppm)
1	2.073
2	1.515
4	1.355
6	1.296
8	1.564
10	1.204

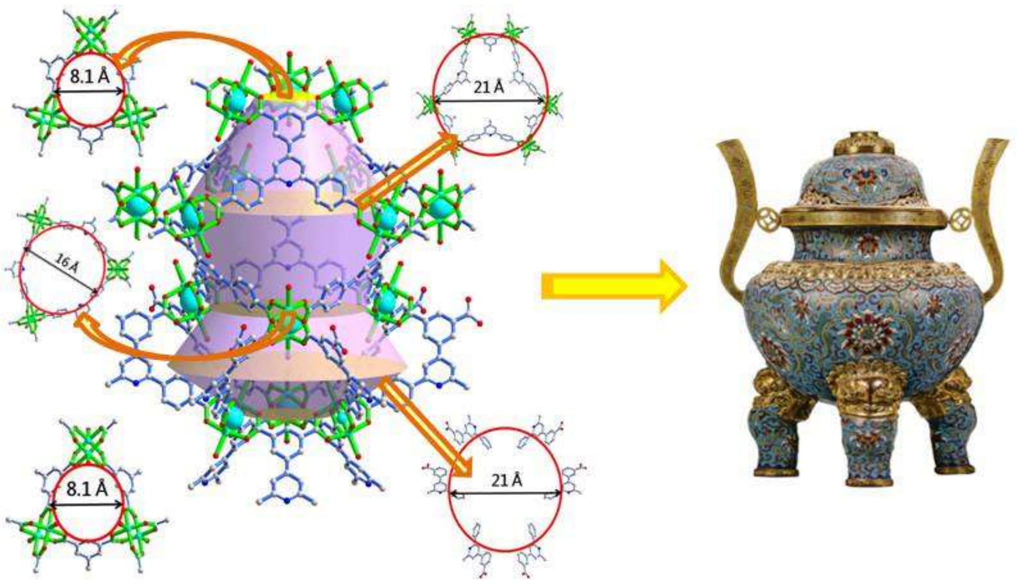

Figure S1. The windows and internal pore size of compound **1**. The shape of [Cu_{30}] cage looks like a censer.

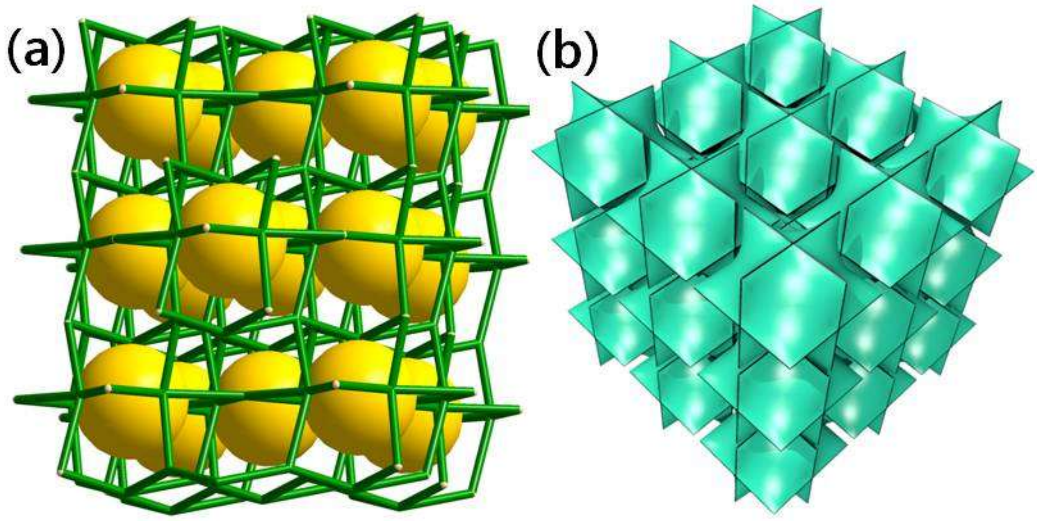

Figure S2. (a) The topology of framework **1**, and yellow balls represent [Cu_{30}] cages; (b) Tiling representations of [Cu_{30}] cage in **1**.

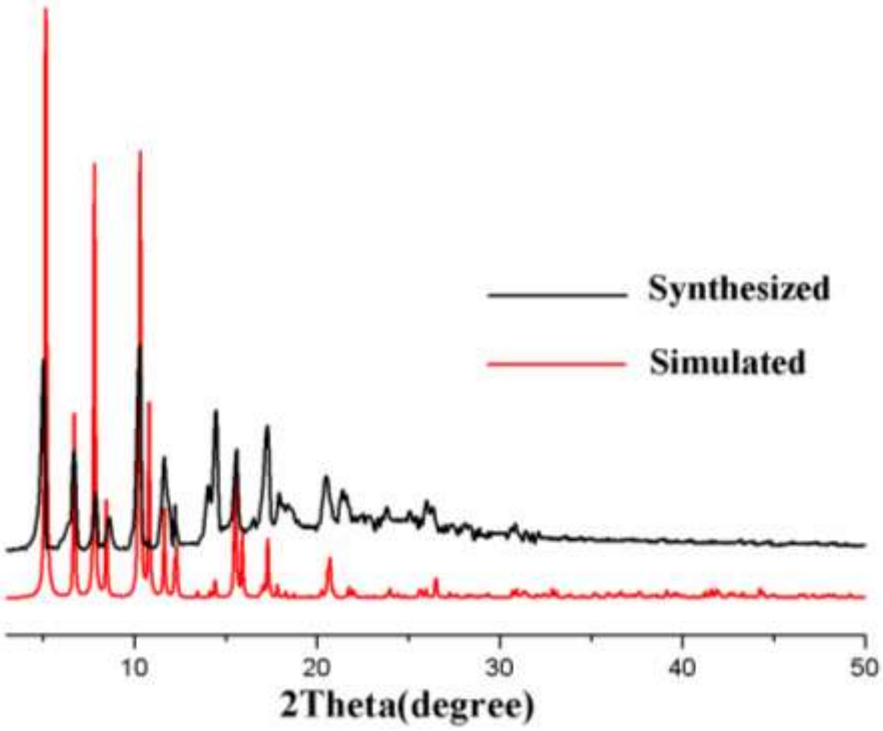

Figure S3. Powder XRD of simulated **1** (red) and as-synthesized **1** (black).

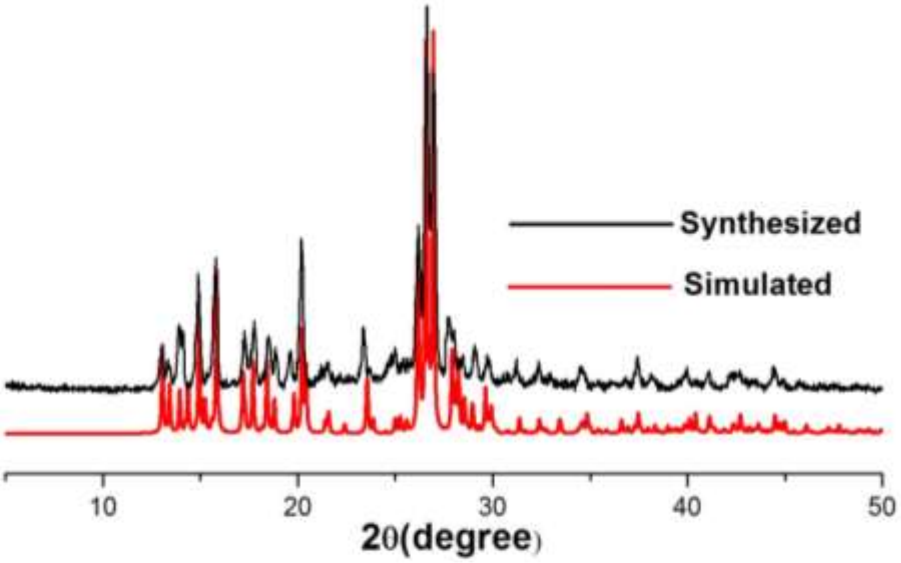

Figure S3. Powder XRD of simulated **H_4BCP** (red) and as-synthesized **H_4BCP** (black).

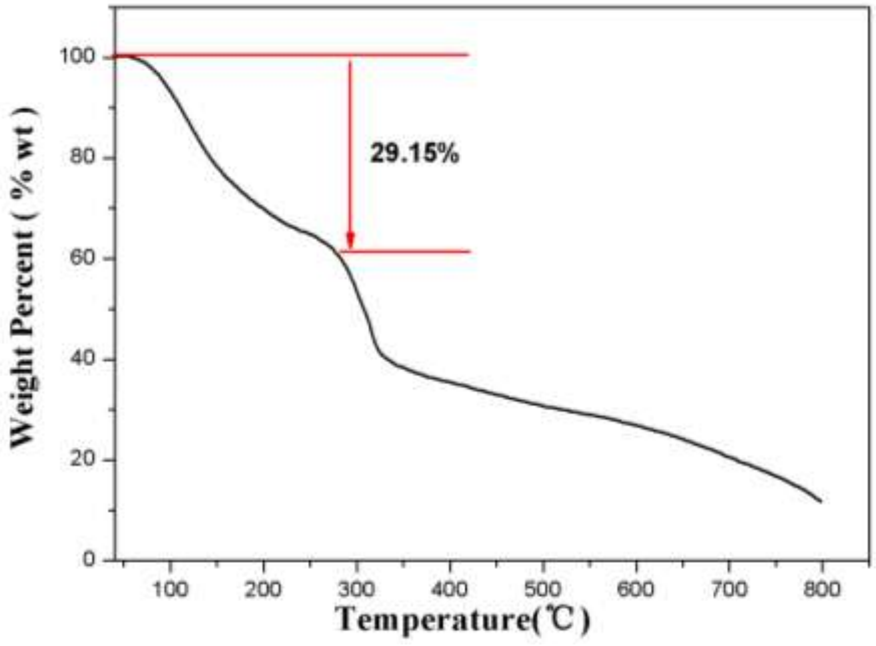

Figure S4. Thermogravimetric analyses curve of **1**, the weight loss of 29.15% attributes to the loss of coordinated H_2O and lattice DMF molecules, which is close to the calculated value (29.56%).

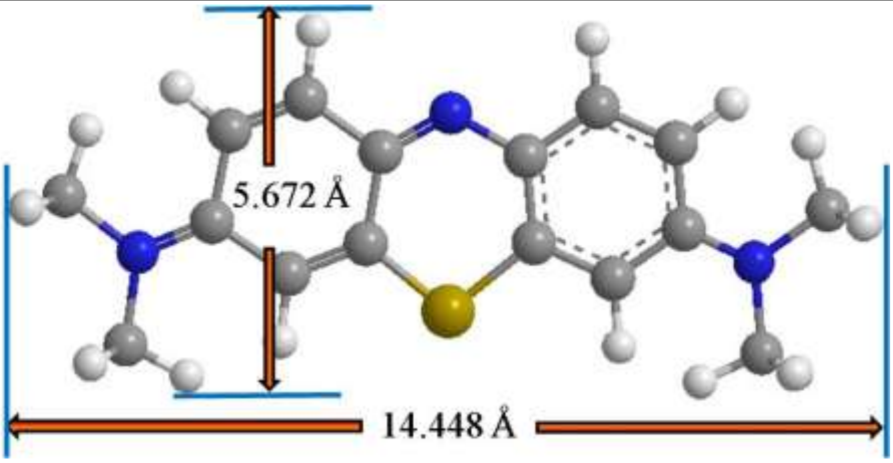

Figure S5. The size of MB molecule.

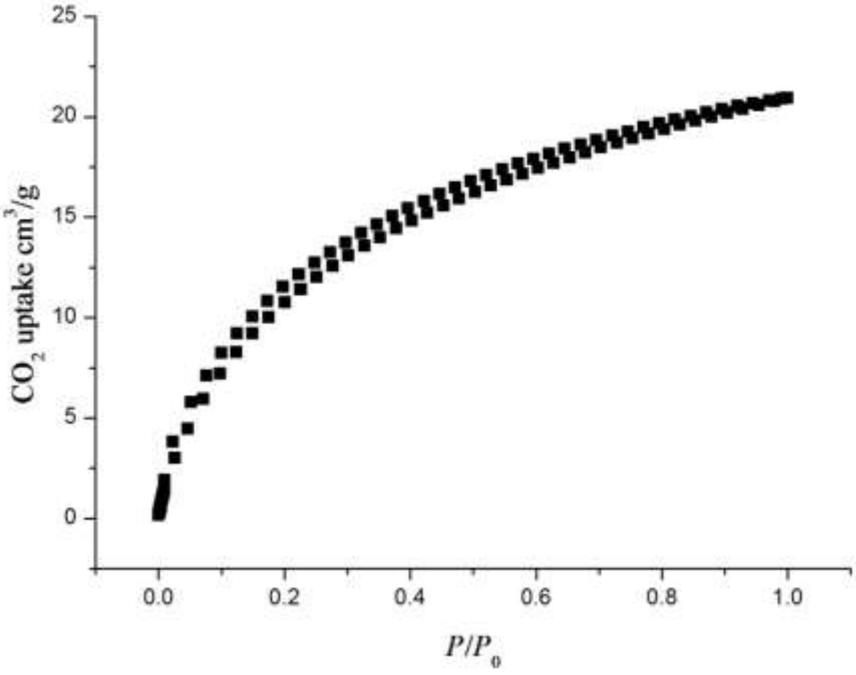

Figure S6. CO_2 adsorption/desorption of compound **1** (293 K).

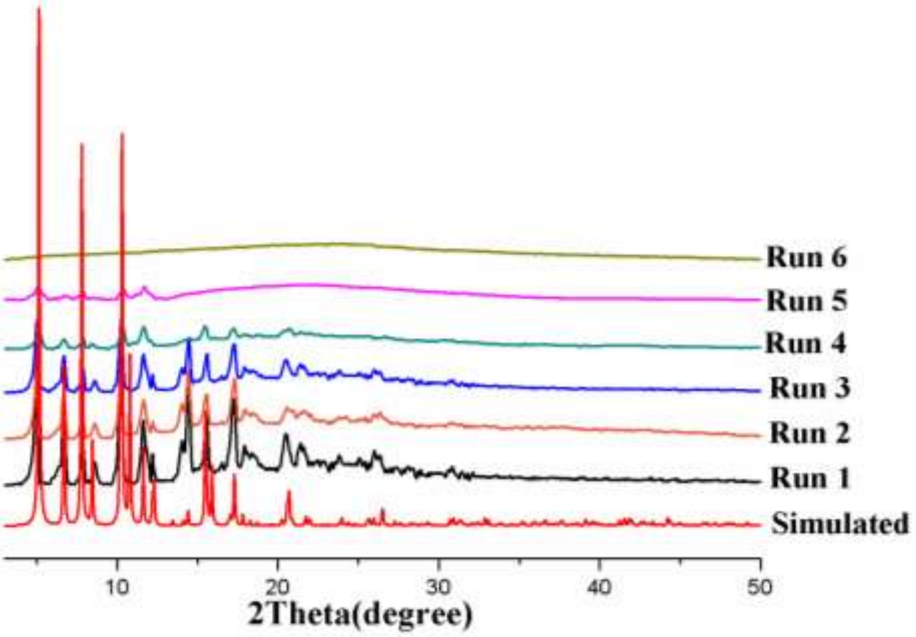

Figure S7. The PXRD patterns of **1** after six recyclings.

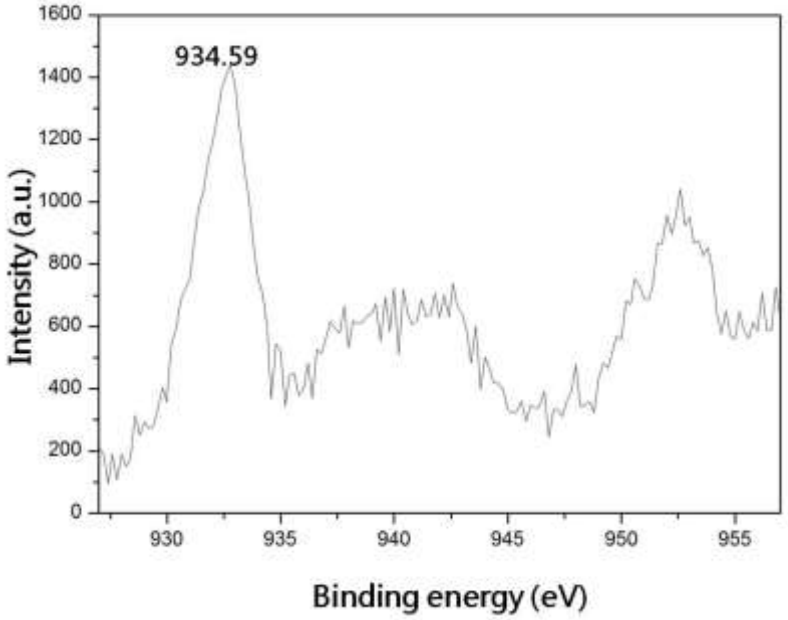

Figure S8. XPS spectrum of Cu in used compound **1**. The significant peak at 934.59 eV (Cu $2P^{3/2}$) and shakeup features are the characteristics of Cu(II).[5]

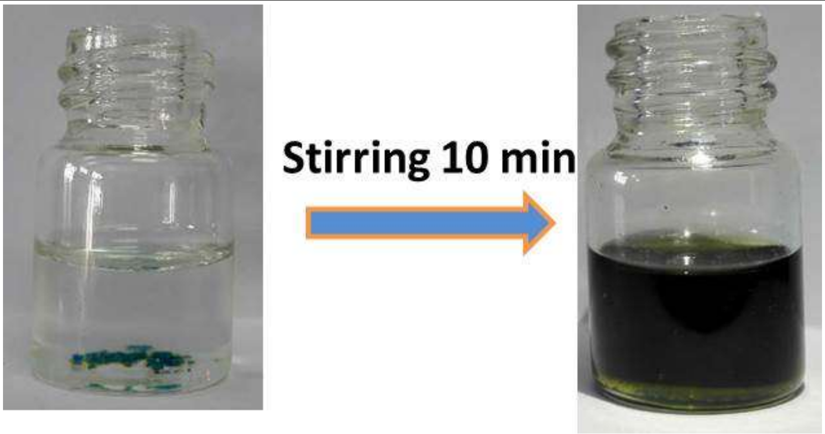

Figure S9. The change of $Cu(NO_3)_3$ and TBAB in1-ethyl-2-phenylaziridine after 10 min stirring. The explorations of the solubility of copper salts ($Cu(OAc)_2$, $Cu(NO_3)_2$, $CuSO_4$) were carried out, and $Cu(NO_3)_2$ was selected as a representative to explain in details. Firstly, $Cu(NO_3)_2$ and TBAB were added into 1-ethyl-2-phenylaziridine. As shown in below picture, initially the solubility of copper salts was low, then, more and more copper salts were dissolved in 1-ethyl-2-phenylaziridine after stirring. Ten minutes later, solid copper salts were not observed, and the color becomes brown, indicating that copper salts have good solubility.

The ^{1}H NMR and ^{13}C NMR charts for H_4BCP

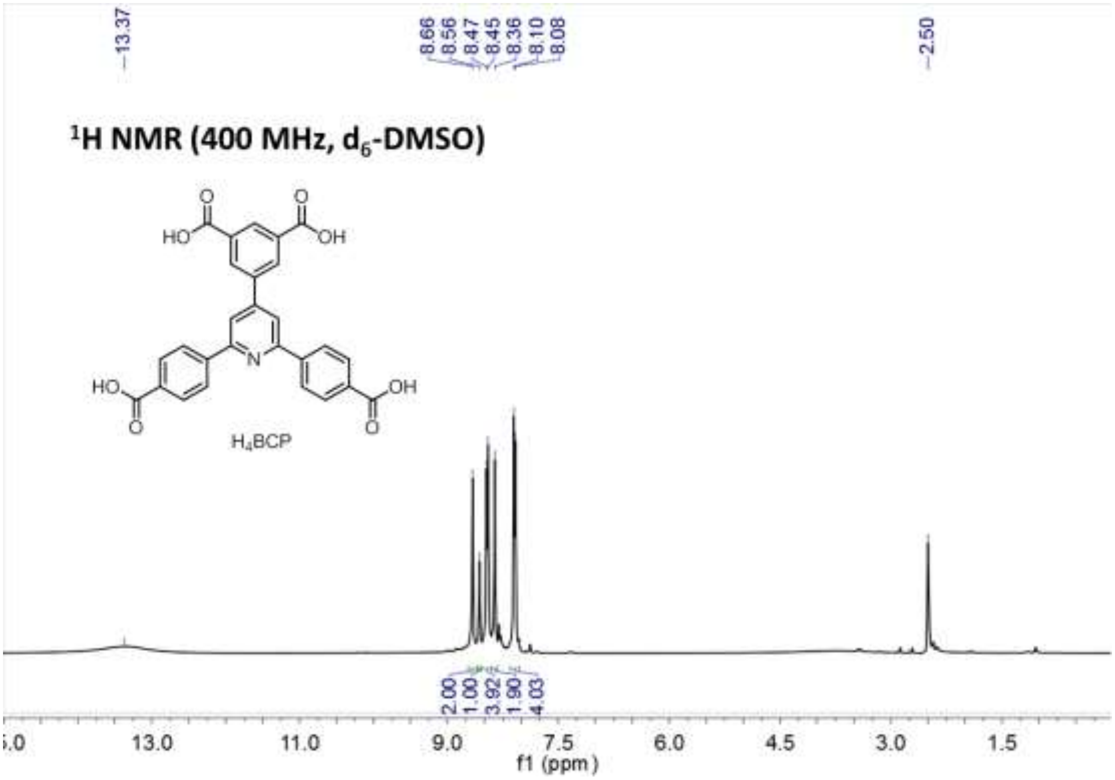

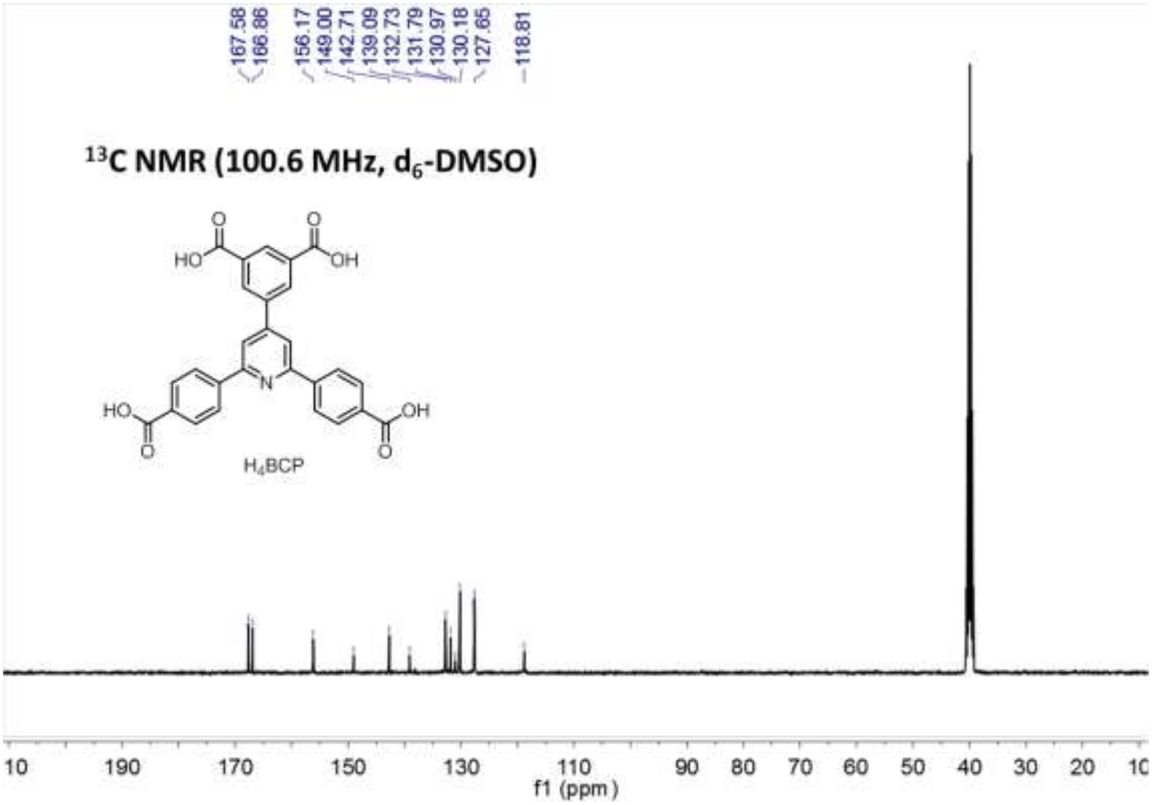

General procedure for the preparation of aziridines

Scheme S3

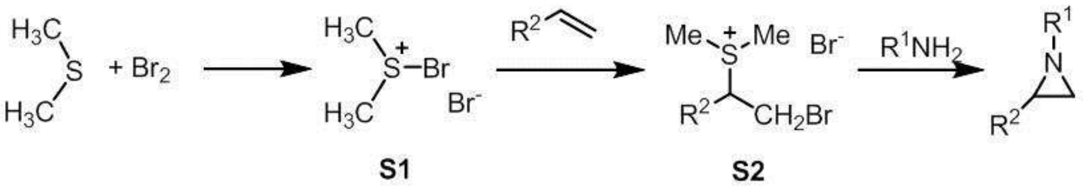

Typical procedure for the synthesis of aziridines was described as following: First step, 0.2 mol bromine (32.0 g) in dry CH_2Cl_2 (40 mL) slowly dropped to 40 mL CH_2Cl_2 solution of dimethyl sulfide (12.4 g, 0.2 mol) under ice-salt baths. Light orange crystals of bromodimethyl sulfonium bromide gradually generated during the process, and the orange crystals **S1** were completely obtained and collected by filtration after the addition of bromine.

Then the products washed with dry diethyl ether and dried under vacuum. Yield: 80%, Mp 80 ^{o}C (dec).

Next step, olefin (160 mmol) slowly dropped to the 160 mL CH_3CN solution of **S1** (35.56 g, 160 mmol) under ice-salt baths. White solid gradually generated during the process, and the solution continued stirring for 10 min. The crystals **S2** was collected by filtration, and dried under vacuum. Yield: 32-38.6 %, Mp 80 ^{o}C (dec).

Last step, a solution of amine (20-50 mmol) slowly dropped to a stirred solution of compound **S2** (10 mmol) in water (20 mL) at room temperature, and the resulting mixture continued stirring overnight. Then the mixture slowly dropped into 20 mL of saturated brine, extracted with diethyl ether (3×20 mL), dried with anhydrous $MgSO_4$ overnight and evaporated under reduced pressure. Aziridine was obtained by distillation under reduced pressure. Yield: 85-100 %.

Characterization data of aziridines

1-Ethyl-2-phenylaziridine

^{1}H NMR (400 MHz, $CDCl_3$) δ 1.17 (t, ^3J = 9.6 Hz, 3 H, CH_2CH_3), 1.65 (d, ^2J = 8.8 Hz, 1 H, NCH_2CH), 1.89 (d, ^2J = 4.4 Hz, 1 H, NCH_2CH), 2.30 (dd, ^3J = 4.4 Hz, ^3J = 4.8 Hz, 1 H, NC*H*Ph), 2.44 (q, ^3J = 9.6 Hz, 2 H, NCH_2CH_3), 7.18-7.31 (m, 5 H, Ar*H*); ESI-MS calcd for $C_{10}H_{13}N$ 147.10, found 148.31 $[M + H]^+$.

1-Propyl-2-phenylaziridine

^{1}H NMR (400 MHz, $CDCl_3$) δ 0.95 (t, ^3J = 10.0 Hz, 3 H, CH_2CH_3), 1.60-1.67 (m, 3 H, CH_2CH_2), 1.89 (d, ^2J = 4.0 Hz, 1 H, CH_2CH_2), 2.24-2.33 (m, 2 H, NCH_2CH), 2.43-2.51 (m, 1 H, NC*H*Ph), 7.18-7.31 (m, 5 H, Ar*H*); ESI-MS calcd for $C_{11}H_{15}N$ 161.12, found 162.28 $[M + H]^+$

1-Butyl-2-phenylaziridine

^{1}H NMR (300 MHz, $CDCl_3$) δ 0.91 (t, ^{3}J = 7.2 Hz, 3 H, CH_2CH_3), 1.33-1.45 (m, 2 H, $CH_3CH_2CH_2$), 1.55-1.67 (m, 3 H, $CH_2CH_2CH_2N$), 1.88 (d, ^{2}J =3.3 Hz, 1 H, CH_2CH_2N), 2.27-2.36 (m, 2 H, NCH_2CH), 2.45-2.54 (m, 1 H, NC*H*Ph), 7.17-7.31 (m, 5 H, Ar*H*); ESI-MS calcd for $C_{12}H_{17}N$ 175.14, found 176.38 $[M + H]^+$.

2-(4-Chlorophenyl)-1-ethylaziridine

^{1}H NMR (300 MHz, $CDCl_3$) δ 1.18 (t, ^{3}J = 6.9 Hz, 3 H, CH_2CH_3), 1.65 (d, ^{2}J = 6.6 Hz, 1 H, NCH_2CH), 1.83 (d, ^{2}J = 3.3 Hz, 1 H, NCH_2CH), 2.25-2.46 (m, 3 H, NCH_2CH_3 and NC*H*Ph), 7.15-7.23 (m, 4 H, Ar*H*); ESI-MS calcd for $C_{10}H_{12}NCl$ 181.66, found 182.13 $[M + H]^+$.

1-Ethyl-2-p-tolylaziridine

^{1}H NMR (400 MHz, $CDCl_3$) δ 1.19 (t, ^{3}J = 7.2 Hz, 3 H, CH_2CH_3), 1.62 (d, ^{2}J = 6.4 Hz, 1 H, NCH_2CH), 1.86 (d, ^{2}J = 3.2 Hz, 1 H, NCH_2CH), 2.26 (dd, ^{3}J = 3.6 Hz, ^{3}J = 3.2 Hz, 1 H, NC*H*Ph), 2.31 (s, 3 H, $PhCH_3$), 2.37-2.48 (m, 2 H), 7.09-7.15 (m, 4 H, Ar*H*); ESI-MS calcd for $C_{11}H_{15}N$ 161.24, found 162.20 $[M + H]^+$.

Characterization data of oxazolidinones

3-Ethyl-5-phenyloxazolidin-2-one

Colorless liquid; ^{1}H NMR (300 MHz, $CDCl_3$) δ 1.17 (t, ^{3}J = 7.2 Hz, 3 H, CH_2CH_3), 3.29-3.45 (m, 3 H, NCH_2CH_3 and OC*H*Ph), 3.92 (t, ^{3}J = 8.7 Hz, 1 H, NCH_2CH), 5.48 (t, ^{3}J = 7.8 Hz, 1 H, NCH_2CH), 7.34-7.42 (m, 5 H, Ar*H*); ^{13}C NMR (75 MHz, $CDCl_3$) δ 12.4, 38.8, 51.5, 74.2, 125.4, 128.6, 128.8, 138.8, 157.5; ESI-MS calcd for $C_{11}H_{13}NO_2$ 191.09, found 192.29 $(M + H)^+$, 214.38 $(M + Na)^+$,405.01 $(2 M + Na)^+$.

3-Ethyl-4-phenyloxazolidin-2-one

Colorless liquid; ^{1}H NMR (400 MHz, CDCl$_3$) δ 1.05 (t, ^{3}J = 7.2 Hz, 3 H, CH$_2$CH_3), 2.79-2.88 (m, 1 H, NCH_2CH$_3$), 3.48-3.57 (m, 1 H, NCH_2CH$_3$), 4.10 (t, ^{3}J = 8.0 Hz, 1 H, NCHPh), 4.62 (t, ^{3}J = 8.8 Hz, 1 H, OCH_2CH), 4.81 (t, ^{3}J = 7.2 Hz, 1 H, OCH_2CH), 7.30 7.44 (m, 5 H, ArH); ^{13}C NMR (75MHz, CDCl$_3$) δ 12.1, 36.8, 59.3, 69.7, 126.9, 129.0, 129.2, 137.8, 158.1; ESI-MS calcd for $C_{11}H_{13}NO_2$ 191.09, found 192.29 (M + H) $^{+}$, 214.38 (M + Na) $^{+}$, 405.01 (2M + Na) $^{+}$.

3-Propyl-5-phenyloxazolidin-2-one

Colorless liquid; ^{1}H NMR (300 MHz, CDCl$_3$) δ 0.91 (t, ^{3}J=7.2 Hz, 3 H, CH$_2$CH_3), 1.52-1.61 (m, 2 H, CH$_3$CH_2), 3.18-3.31 (m, 2 H, CH$_2$CH_2N) 3.40 (t, ^{3}J=8.0 Hz, 1 H, OCH_2CH), 3.90 (t, ^{3}J=8.8 Hz, 1 H, OCH_2CH), 5.46 (t, ^{3}J=8.0 Hz, 1 H, NCHPh), 7.31-7.37 (m, 5 H, ArH); ^{13}C NMR (75 MHz, CDCl$_3$) δ 10.7, 20.3, 45.5, 51.8, 74.0, 125.2, 128.4, 128.5, 138.7, 157.6; ESI-MS calcd for $C_{12}H_{15}NO_2$ 205.11, found 206.30 (M + H) $^{+}$, 228.30 (M + Na)$^{+}$, 433.04 (2M + Na)$^{+}$.

3-Butyl-5-phenyloxazolidin-2-one

Colorless liquid; ^{1}H NMR (300 MHz, CDCl$_3$) δ 0.94 (t, ^{3}J=7.2 Hz, 3 H, CH$_2$CH_3), 1.31-140 (m, 2 H, CH$_3$CH_2), 1.51-1.58 (m, 2 H, CH$_2$CH_2CH$_2$), 3.23-3.38 (m, 2 H, NCH_2CH$_2$) 3.43 (t, ^{3}J=8.0 Hz, 1 H, OCH_2CH), 3.92 (t, ^{3}J=8.8 Hz, 1 H, OCH_2CH), 5.49 (t, ^{3}J=8.0 Hz, 1 H, NCHPh), 7.28-7.42 (m, 5 H, ArH); ^{13}C NMR (75 MHz, CDCl$_3$) δ 13.4, 19.5, 29.1, 43.6, 51.8, 74.1, 125.2, 128.4, 128.5, 138.7, 157.7; ESI-MS calcd for $C_{13}H_{17}NO_2$ 219.13, found 220.34 (M + H)$^{+}$, 259.48 (M + K)$^{+}$, 461.05 (2M + Na)$^{+}$.

3-Ethyl-5-(4-chlorophenyl)oxazolidin-2-one

White solid; ^{1}H NMR (400 MHz, CDCl$_3$) δ 1.17 (t, ^{3}J=7.3 Hz, 3 H, CH$_2$CH_3), 3.30-3.43 (m, 2 H, CH$_3$CH_2), 3.69-3.76 (m, 1 H, NCHPh), 3.92 (t, ^{3}J=8.7 Hz, 1 H, OCH_2CH), 5.44 (t, ^{3}J=8.0

Hz, 1 H, OCH_2CH), 7.27-7.38 (m, 4 H, Ar*H*); ^{13}C NMR (100 MHz, $CDCl_3$) δ 12.6, 38.9, 51.5, 73.6, 126.9, 129.1, 134.7, 137.4, 157.4; ESI-MS calcd for $C_{11}H_{12}ClNO_2$ 225.67, found 451.64 $(2M + H)^+$.

3-Ethyl-5-p-tolyloxazolidin-2-one

White solid; ^{1}H NMR (400 MHz, $CDCl_3$) δ 1.18 (t, ^3J=7.3 Hz, 3 H, CH_2CH_3), 1.62 (d, ^3J=6.4 Hz, 1 H, OCH_2CH),1.87 (d, ^3J=3.2 Hz, 1 H, NC*H*Ph), 2.27 (dd, ^3J=6.6 Hz, ^2J=3.2 Hz, 1 H, OCH_2CH), 2.31 (s, 3 H, PhCH_3), 2.36-2.48 (m, 2 H, NCH_2CH$_3$), 7.09- 7.15 (m, 4 H, Ar*H*); ^{13}C NMR (100 MHz, $CDCl_3$) δ 12.6, 21.2, 38.9, 51.6, 74.3, 125.6, 129.5, 135.8, 138.7, 157.7; ESI-MS calcd for $C_{12}H_{15}NO_2$ 205.25, found 206.45 $(M + H)^+$, 411.15 $(2M + H)^+$.

The ^{1}H NMR charts for aziridines

1-Ethyl-2-phenylaziridine

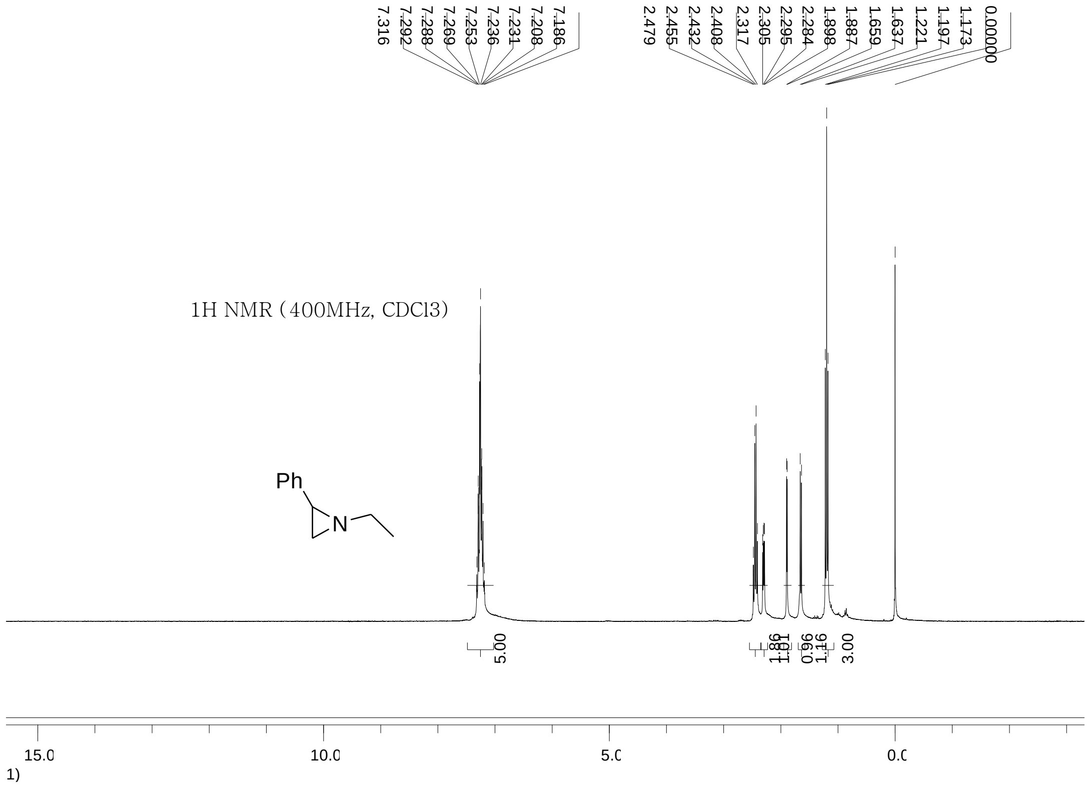

1-Propyl-2-phenylaziridine

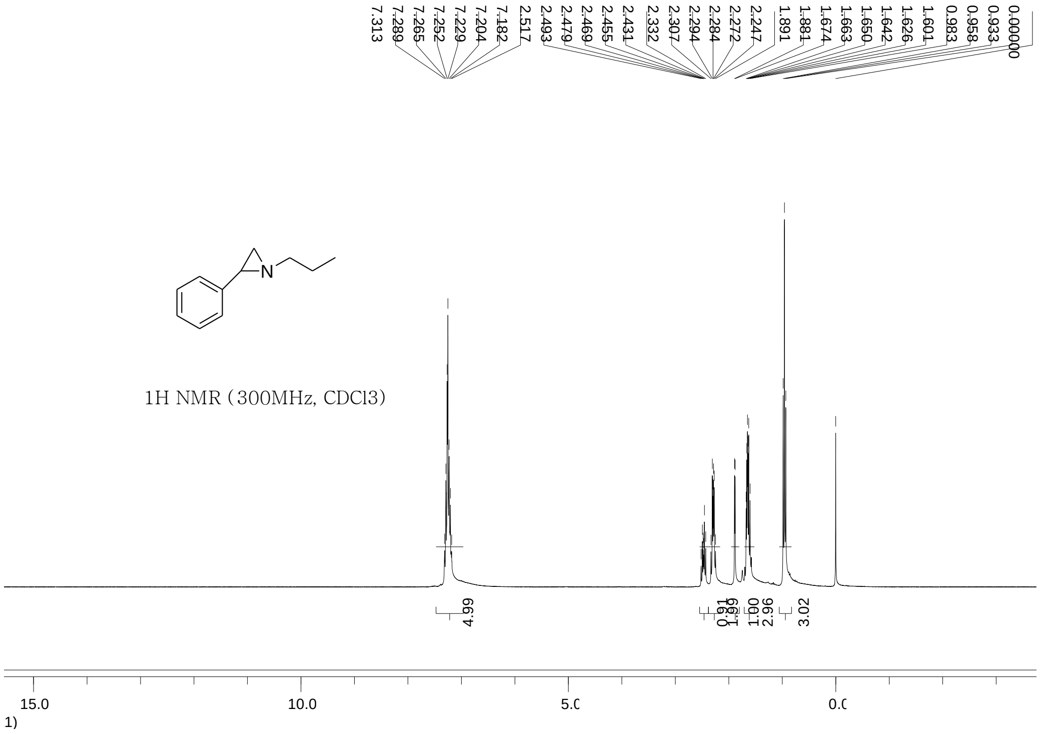

Butyl-2-phenylaziridine

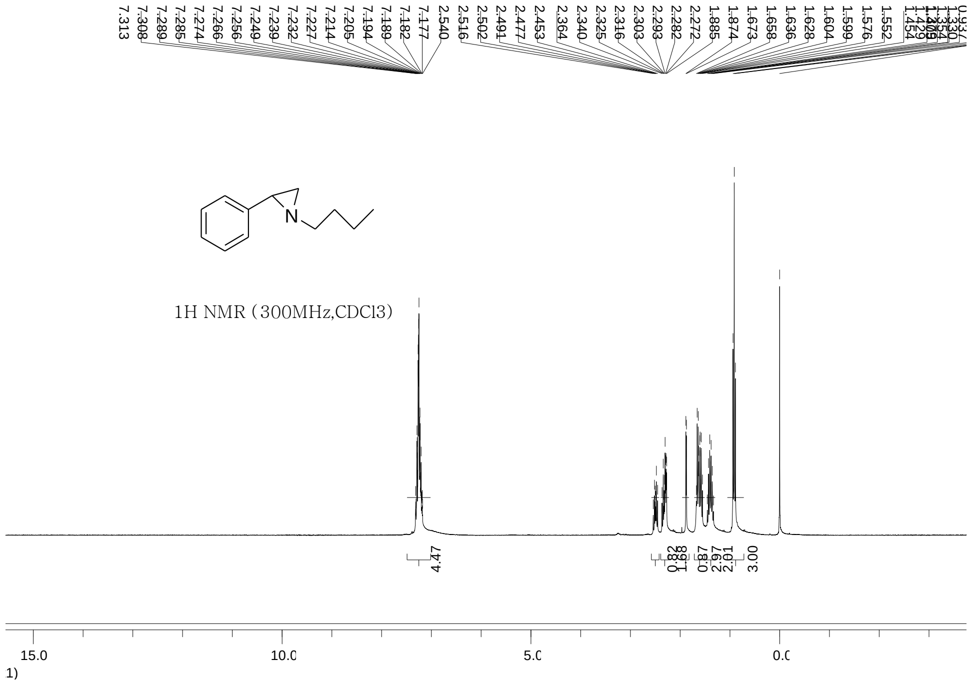

Ethyl-2-p-tolylaziridine

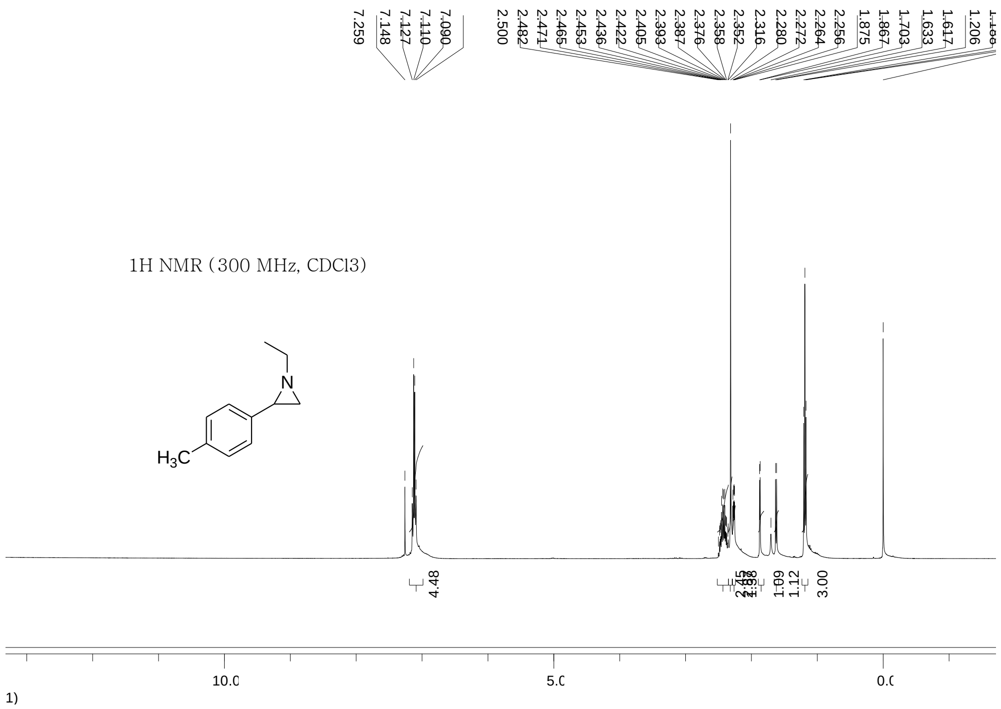

(4-Chlorophenyl)-1-ethylaziridine

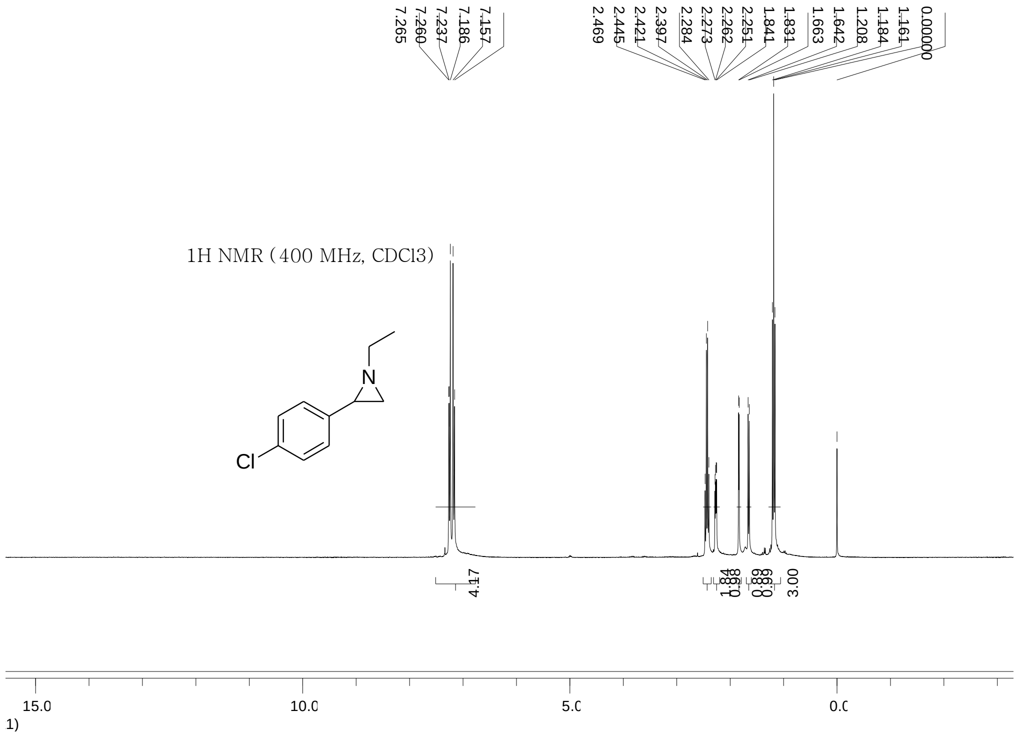

The ^{1}H NMR and ^{13}C NMR charts for oxazolidinones

3-Ethyl-5-phenyloxazolidin-2-one

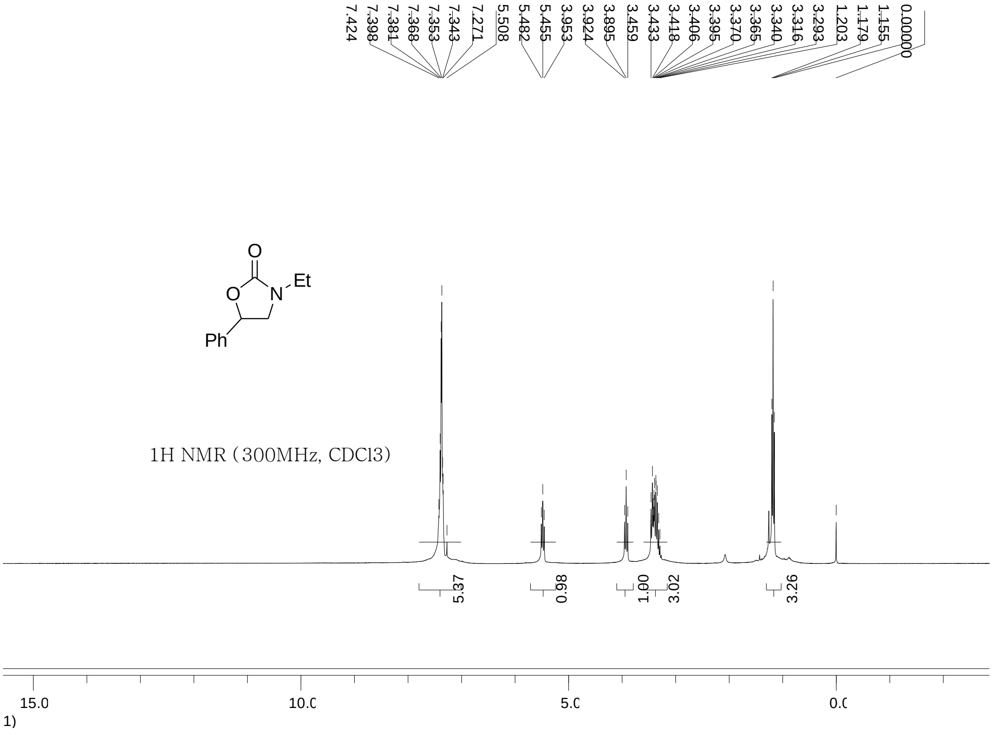

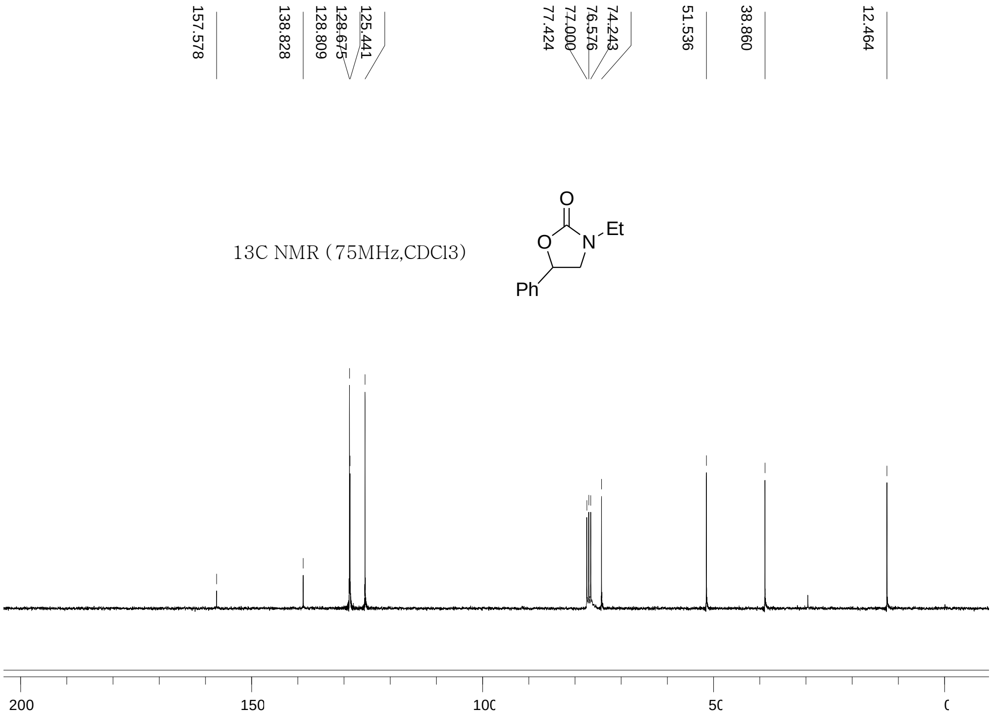

3-Ethyl-4-phenyloxazolidin-2-one

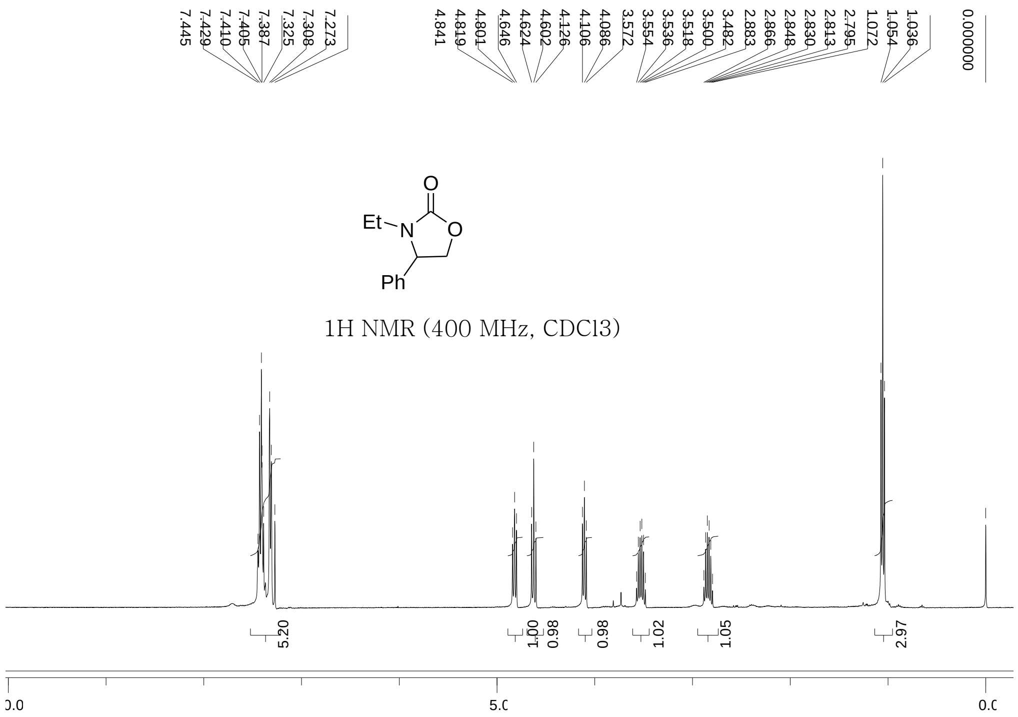

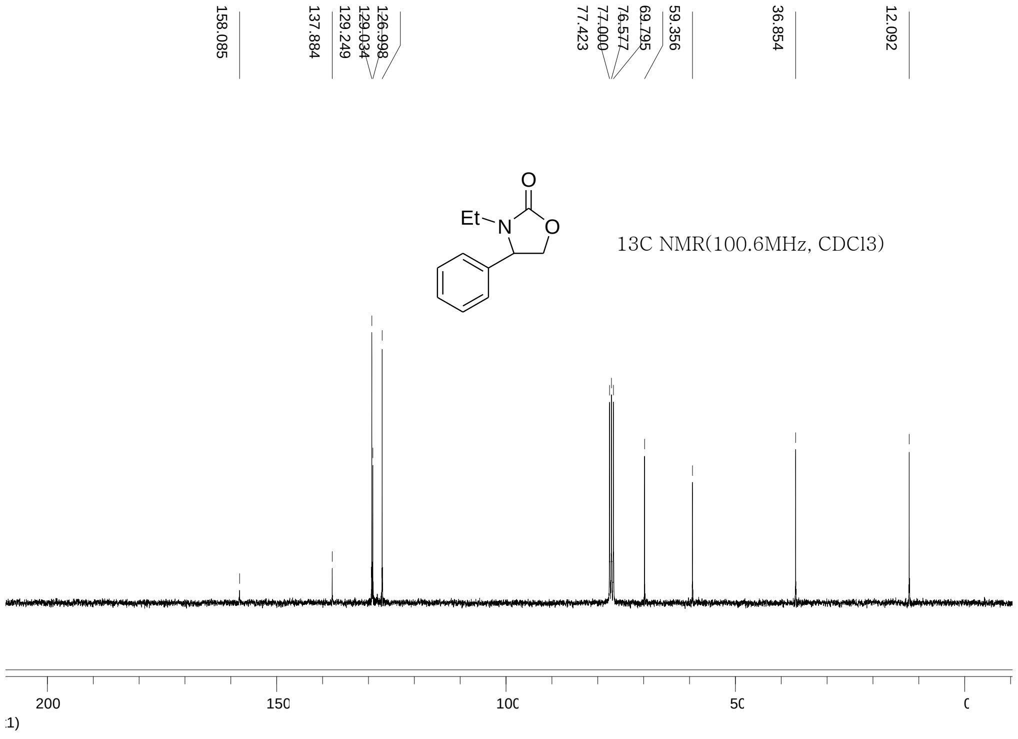

Propyl-5-phenyloxazolidin-2-one

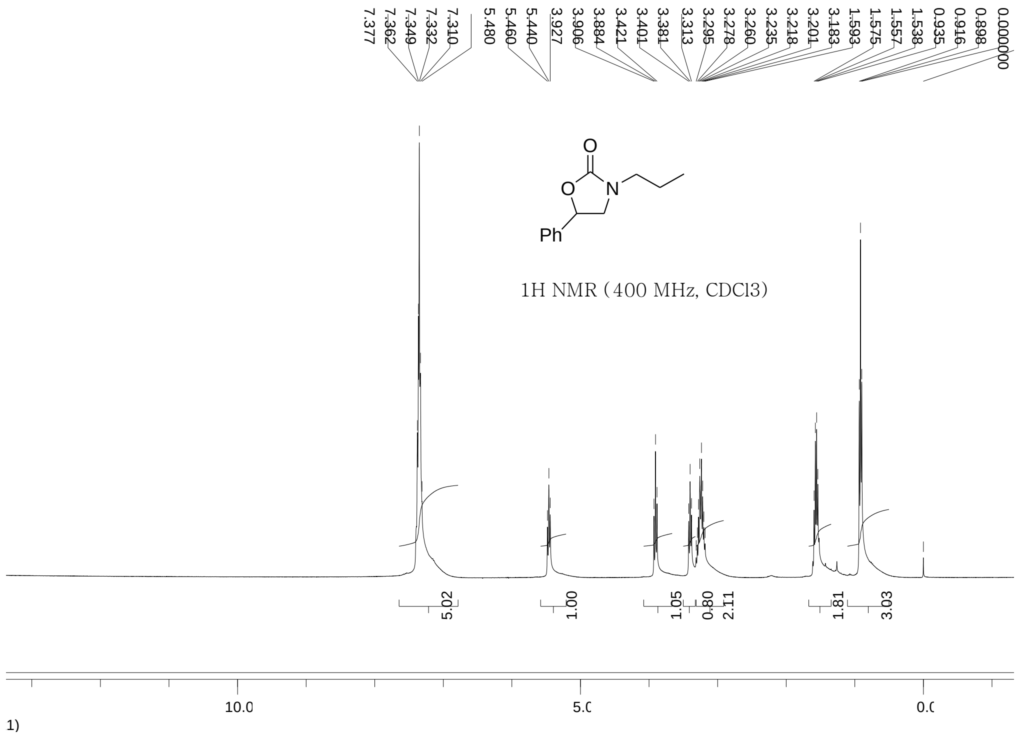

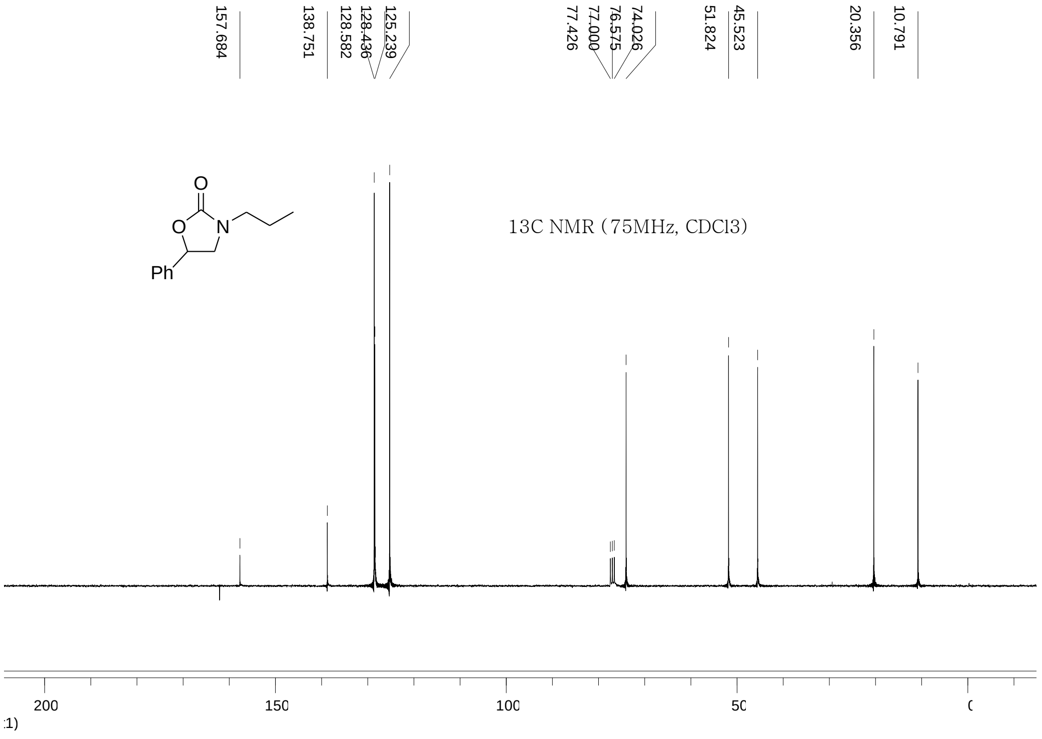

3-Butyl-5-phenyloxazolidin-2-one

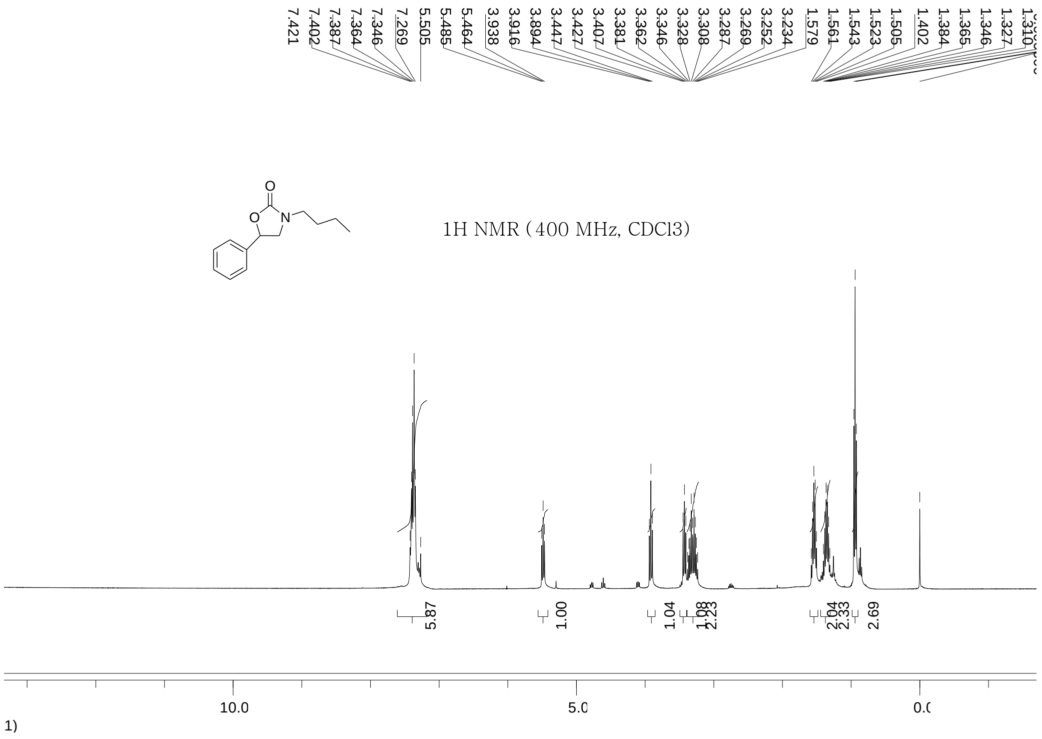

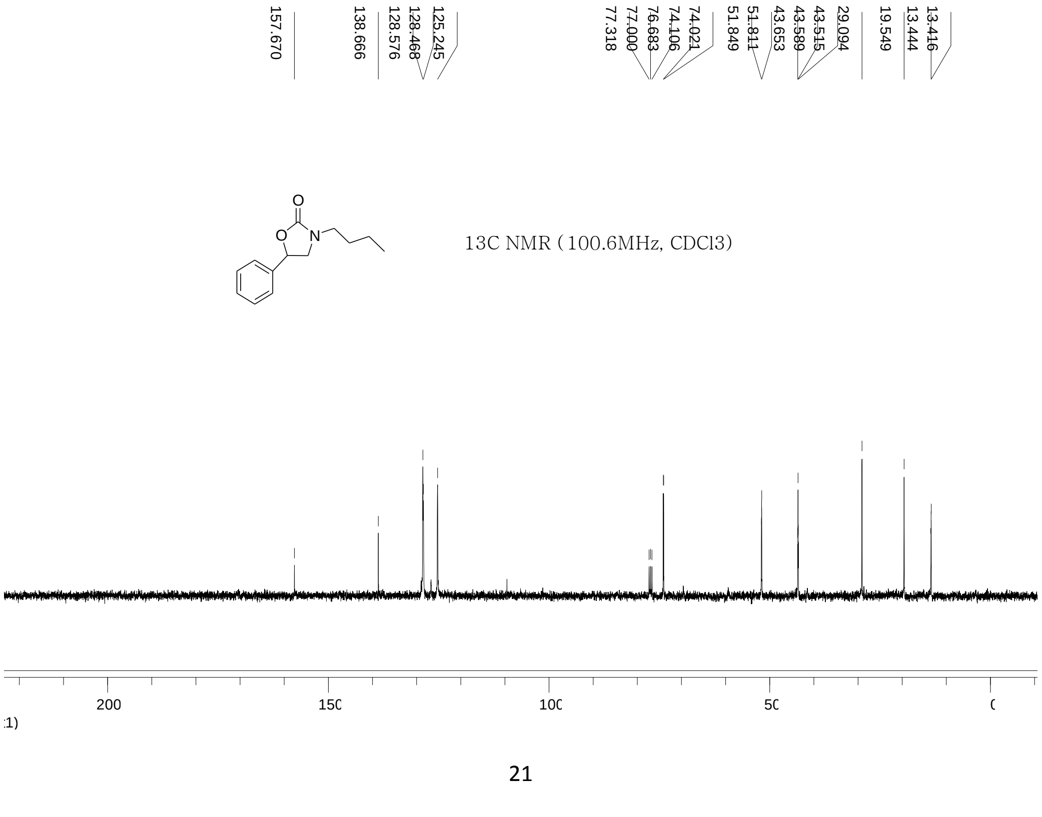

Ethyl-5-(4-chlorophenyl)oxazolidin-2-one

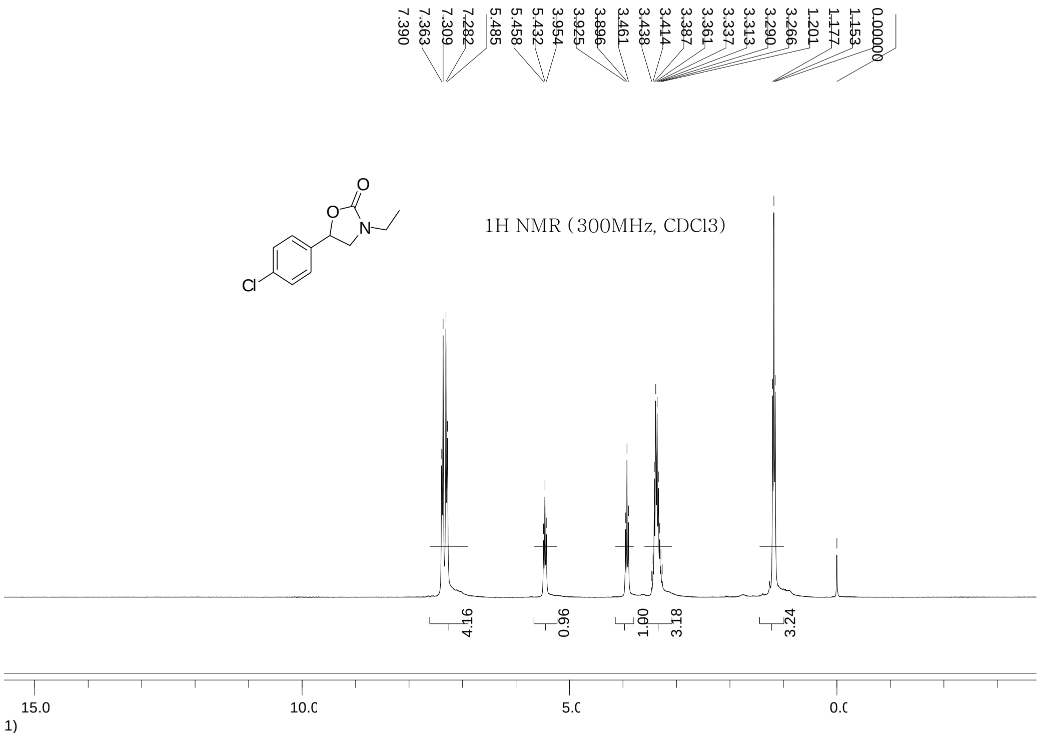

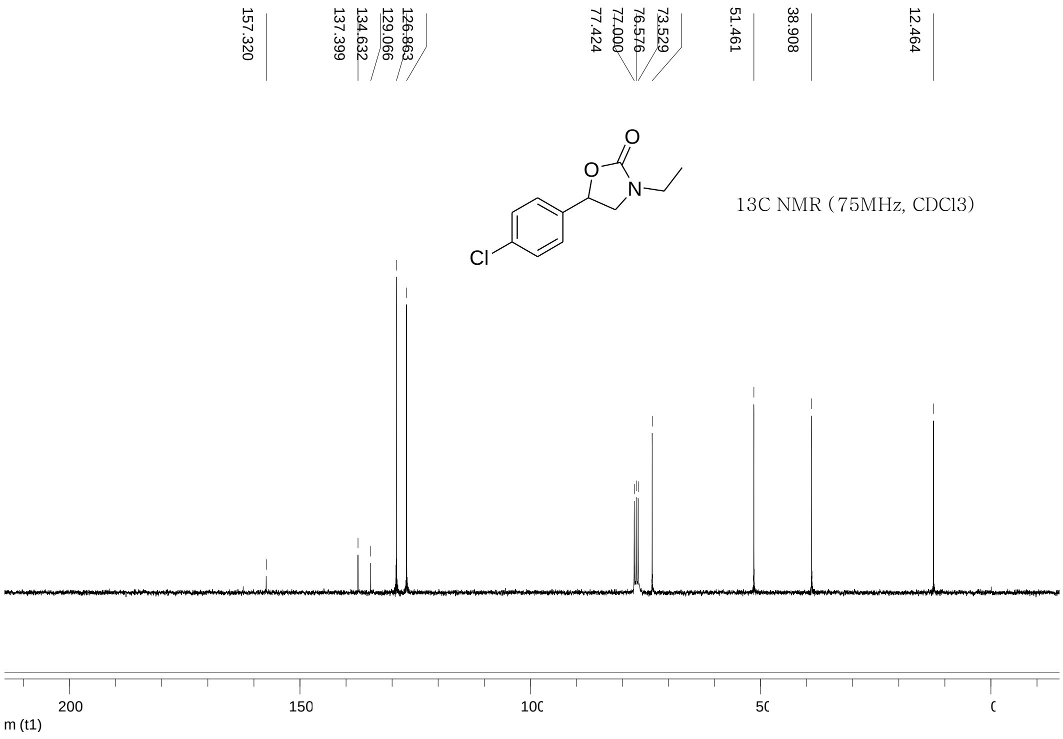

Ethyl-5-p-tolyloxazolidin-2-one

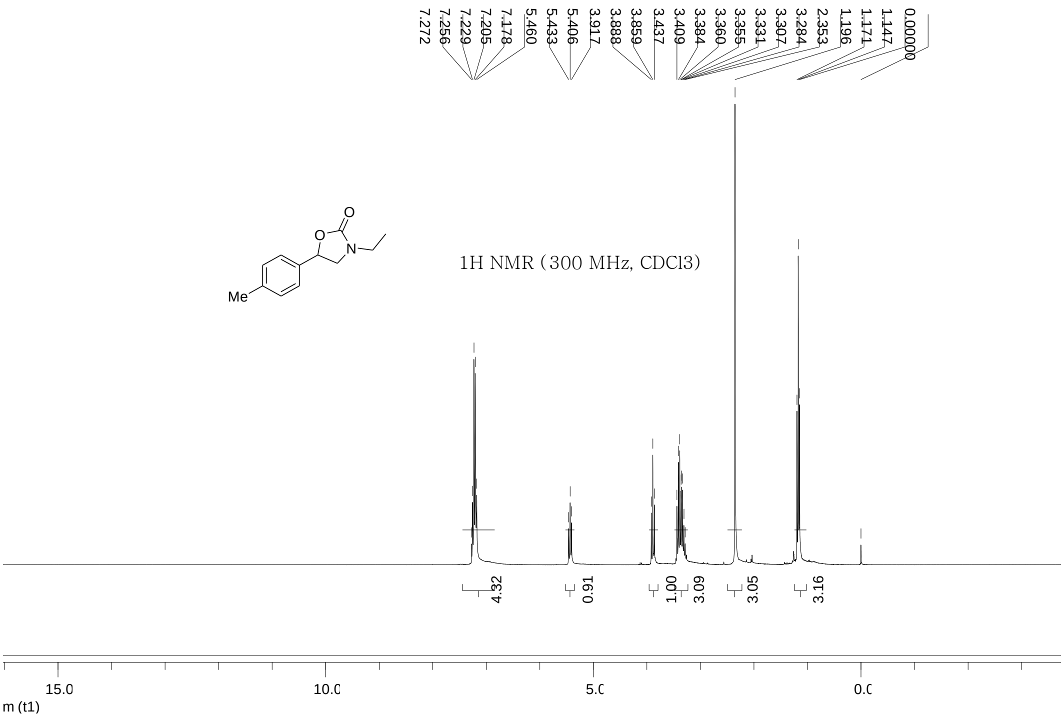

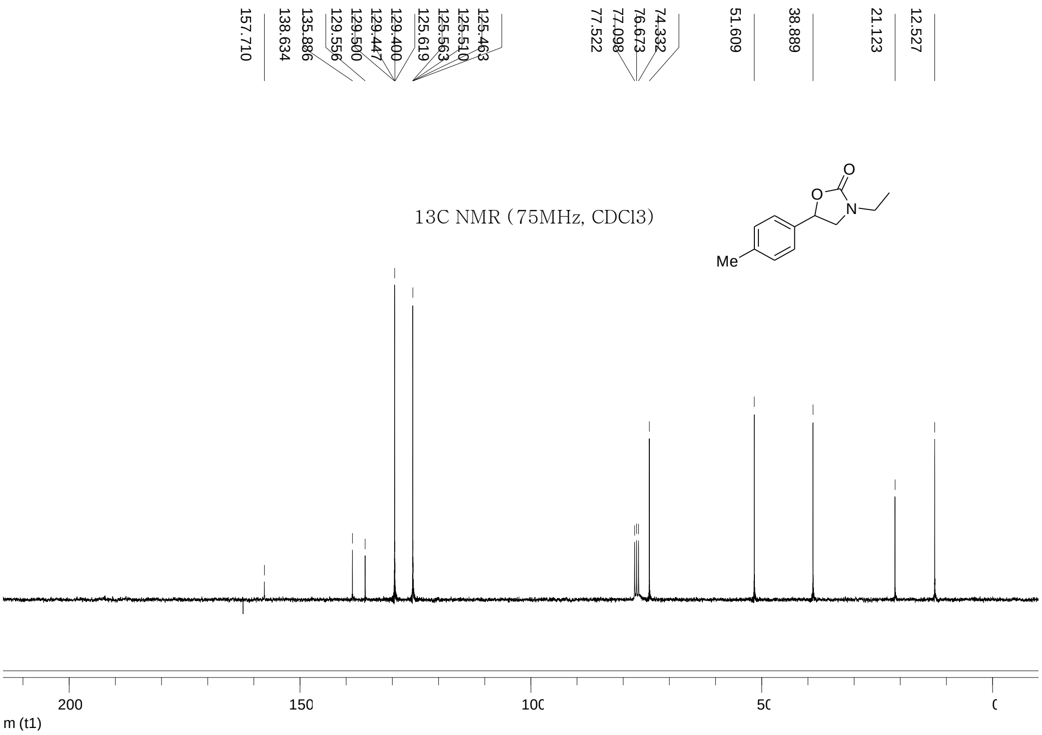

[1] a) Y. Wu, L. N. He, Y. Du, J. Q. Wang, C. X. Miao, W. Li, *Tetrahedron* **2009**, *65*, 6204; b) Y. Du, Y. Wu, A. H. Liu, L. N. He, *J. Org. Chem.* **2008**, *73*, 4709.

[2] SAINT^{+}, version 6.22; Bruker AXS: Madison, **2001**.

[3] G. Sheldrick, *Acta Crystallogr., Sect. A: Fundam.* Crystallogr. **2008**, *64*, 112.

[4] Spek, A. L. *PLATON, A Multipurpose Crystallographic Tool*, Utrecht University, Utrecht, The Netherlands, **2001**.

[5] NIST X-ray Photoelectron Spectroscopy Database, http://srdata.nist.gov/xps/.
